# Supplementary material for: TILTomorrow today: dynamic factors predicting changes in intracranial pressure treatment intensity after traumatic brain injury
Source: Sci Rep. 2025 Jan 2;15:95. doi: 10.1038/s41598-024-83862-x (PMC11696189; doi:10.1038/s41598-024-83862-x)
Supplement: Supplementary file 1 — Supplementary Information. [file 41598_2024_83862_MOESM1_ESM.pdf]

Supplementary information for

TILTomorrow today: dynamic factors predicting changes in intracranial pressure treatment intensity after traumatic brain injury

Shubhayu Bhattacharyay, Florian D van Leeuwen, Erta Beqiri, Cecilia Åkerlund, Lindsay Wilson, Ewout W Steyerberg, David W Nelson, Andrew I R Maas, David K Menon, Ari Ercole, and the CENTER-TBI investigators and participants

Table of Contents

Supplementary Figures **2**

Supplementary Fig. S1. Flow diagram for patient enrolment. *2*

Supplementary Fig. S2. Distributions of $TIL^{(Basic)}$ in the study population over days of ICU stay. *2*

Supplementary Fig. S3. Distributions of $TIL^{(Basic)}$ in the study population stratified by previous $TIL^{(Basic)}$ score. *3*

Supplementary Fig. S4. Distributions of $TIL^{(Basic)}$ directly preceding/following a change in $TIL^{(Basic)}$. *4*

Supplementary Fig. S5. Population-level ΔTimeSHAP values stratified by pre-transition $TIL^{(Basic)}$ score. *5*

Supplementary Fig. S6. Population-level ΔTimeSHAP values for missing value tokens. *8*

Supplementary Tables **9**

Supplementary Table S1. Manually excluded variables indicating death or withdrawal of life-sustaining treatment. *9*

Supplementary Table S2. Physician-based impression variables. *13*

Supplementary Methods **17**

Supplementary Methods S1. Description of model endpoints and outputs for TILTomorrow. *17*
Post-processing calibration 17

Supplementary Methods S2. Repeated Bootstrap Bias Corrected with Dropping Cross-Validation (BBCD-CV). *19*
Dropout of low-performing hyperparametric configurations 19
Confidence intervals for testing set performance 19

Supplementary Methods S3. Hyperparameter optimisation report. *20*
Summary 20
Overview of tested hyperparameters 20
Tested hyperparameters per model version 20

Supplementary Methods S4. Calculation of Somers' D_{xy}. *25*

Supplementary Methods S5. Explanation of model outputs with Shapley value estimations. *27*
Shapley values 27
KernelSHAP 27
TimeSHAP and ΔTimeSHAP 28

Supplementary References **30**

SUPPLEMENTARY FIGURES

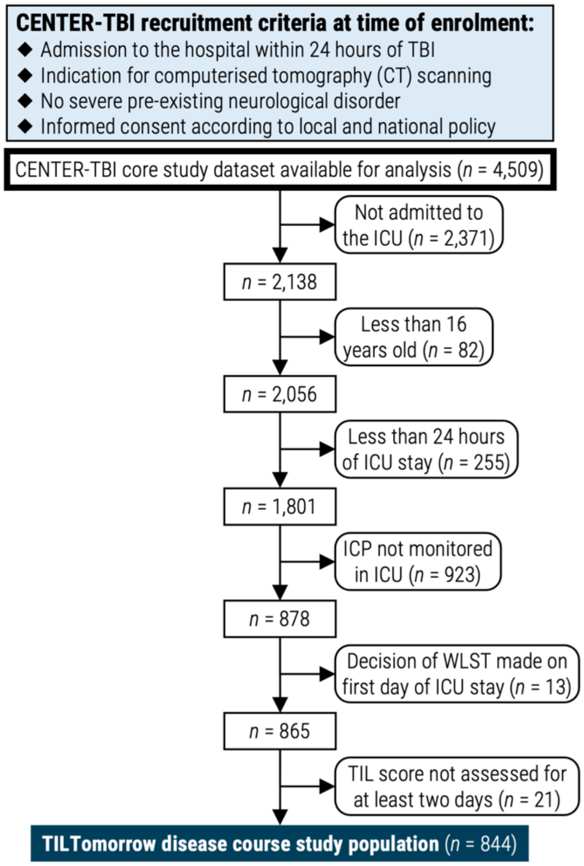

Supplementary Fig. S1. Flow diagram for patient enrolment. Abbreviations: CENTER-TBI=Collaborative European NeuroTrauma Effectiveness Research in TBI, ICP=intracranial pressure, ICU=intensive care unit, TBI=traumatic brain injury, TIL=Therapy Intensity Level scale, WLST=withdrawal of life-sustaining therapies.

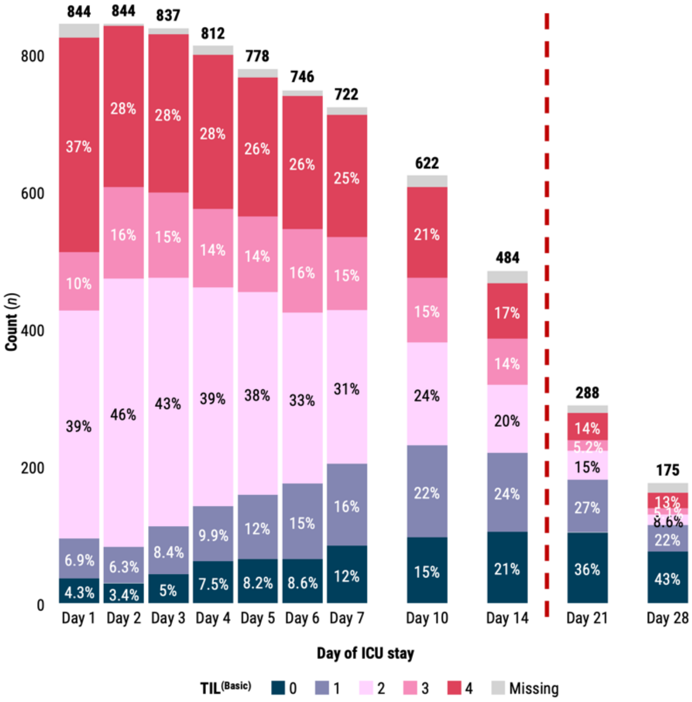

Supplementary Fig. S2. Distributions of TIL$^{(Basic)}$ in the study population over days of ICU stay. Percentages are calculated out of the number of study patients remaining in the ICU at the corresponding day (written above each bar), and percentages which round to 2% or lower are not shown. The days of ICU stay before the vertical, dashed red line were used

for assessment of the TILTomorrow modelling strategy. Abbreviations: ICU=intensive care unit, TIL=Therapy Intensity Level, TIL$^{(Basic)}$=condensed, five-category TIL scale as defined in Table 1.

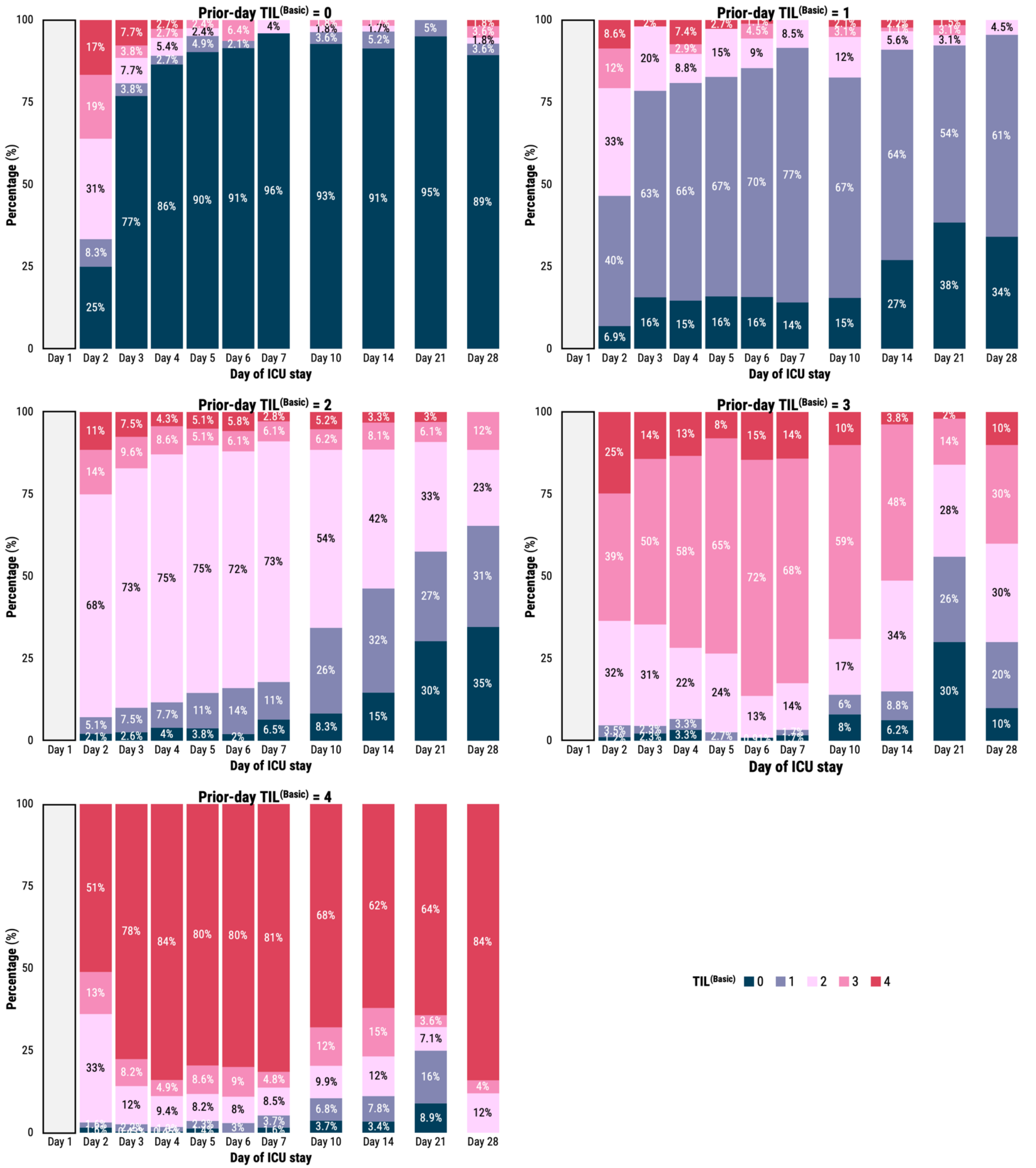

Supplementary Fig. S3. Distributions of TIL$^{(Basic)}$ in the study population stratified by previous TIL$^{(Basic)}$ score. Percentages are calculated out of the number of study patients remaining in the ICU at the corresponding day whose prior-day TIL$^{(Basic)}$ score equalled the score above the panel. Abbreviations: ICU=intensive care unit, TIL=Therapy Intensity Level, TIL$^{(Basic)}$=condensed, five-category TIL scale as defined in Table 1.

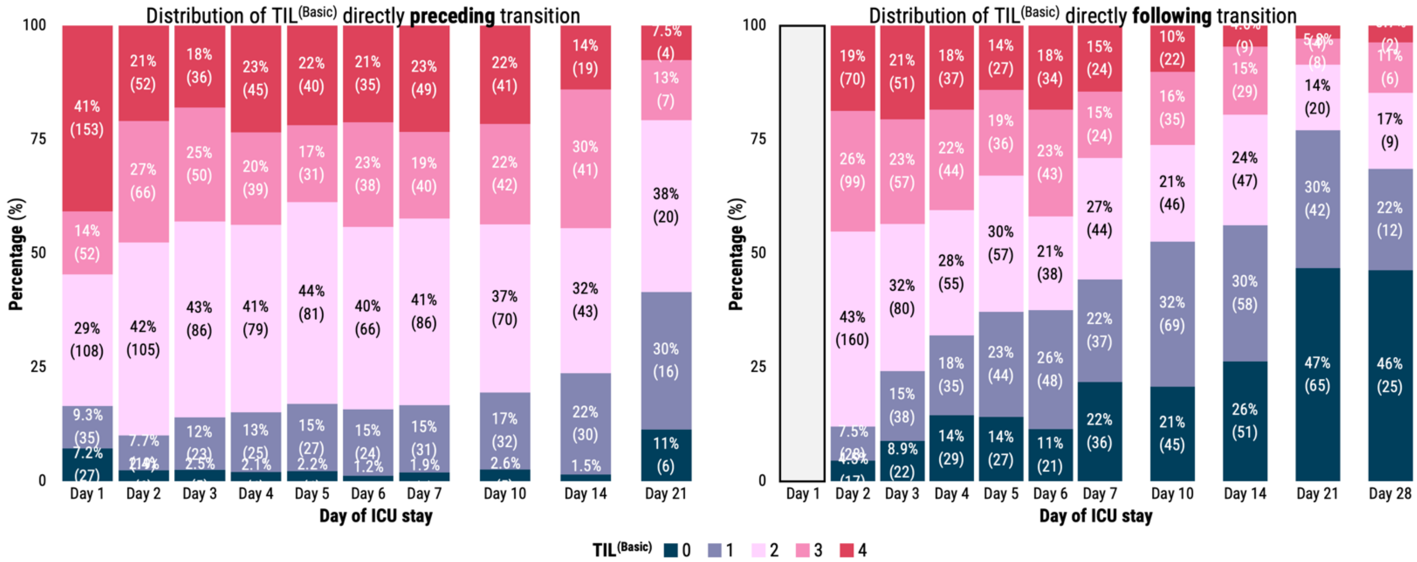

Supplementary Fig. S4. Distributions of TIL$^{(Basic)}$ directly preceding/following a change in TIL$^{(Basic)}$. Percentages are calculated out of the number of study patients who experienced a day-to-day change in TIL$^{(Basic)}$ either directly after (left-hand side) or directly before (right-hand side) the corresponding day of ICU stay. Abbreviations: ICU=intensive care unit, TIL=Therapy Intensity Level, TIL$^{(Basic)}$=condensed, five-category TIL scale as defined in Table 1.

Supplementary Fig. S5. Population-level ΔTimeSHAP values stratified by pre-transition TIL$^{(Basic)}$ score. Legend provided at end of figure (p. 7).

a

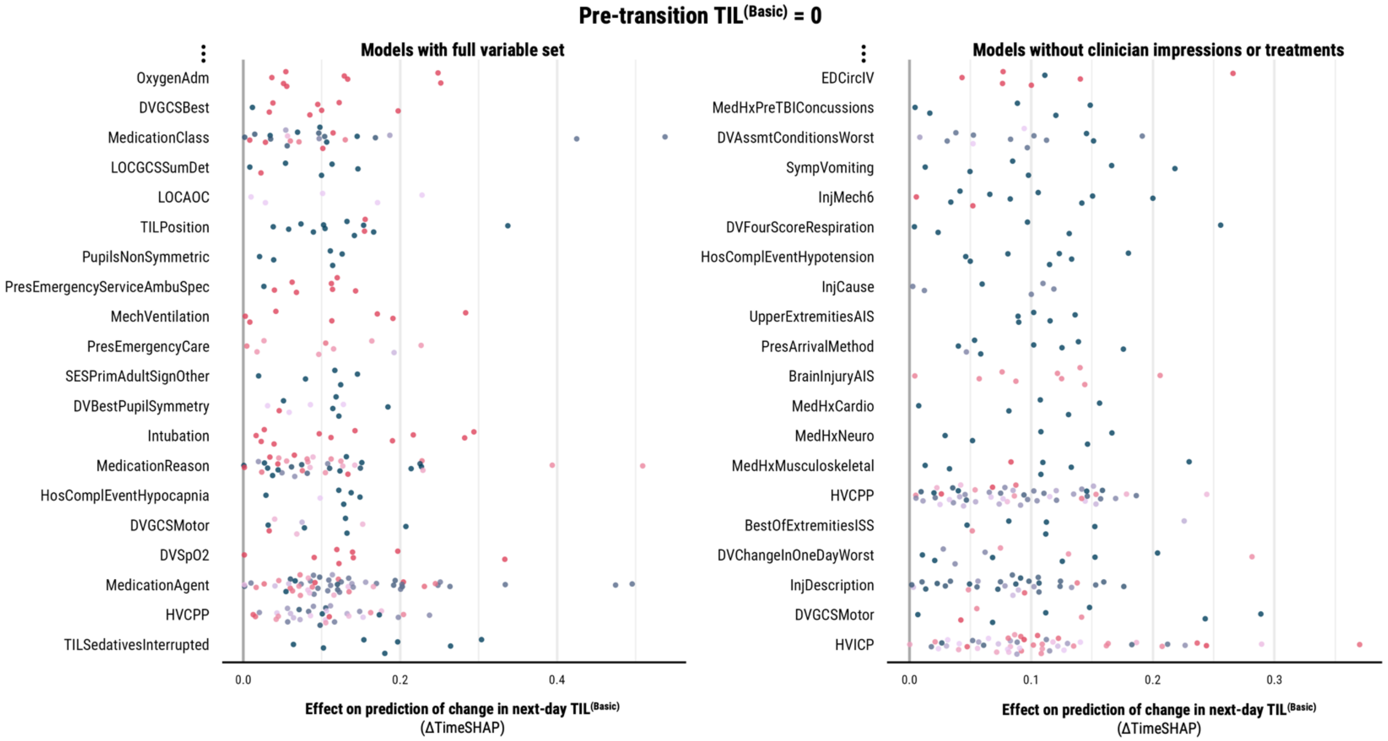

b

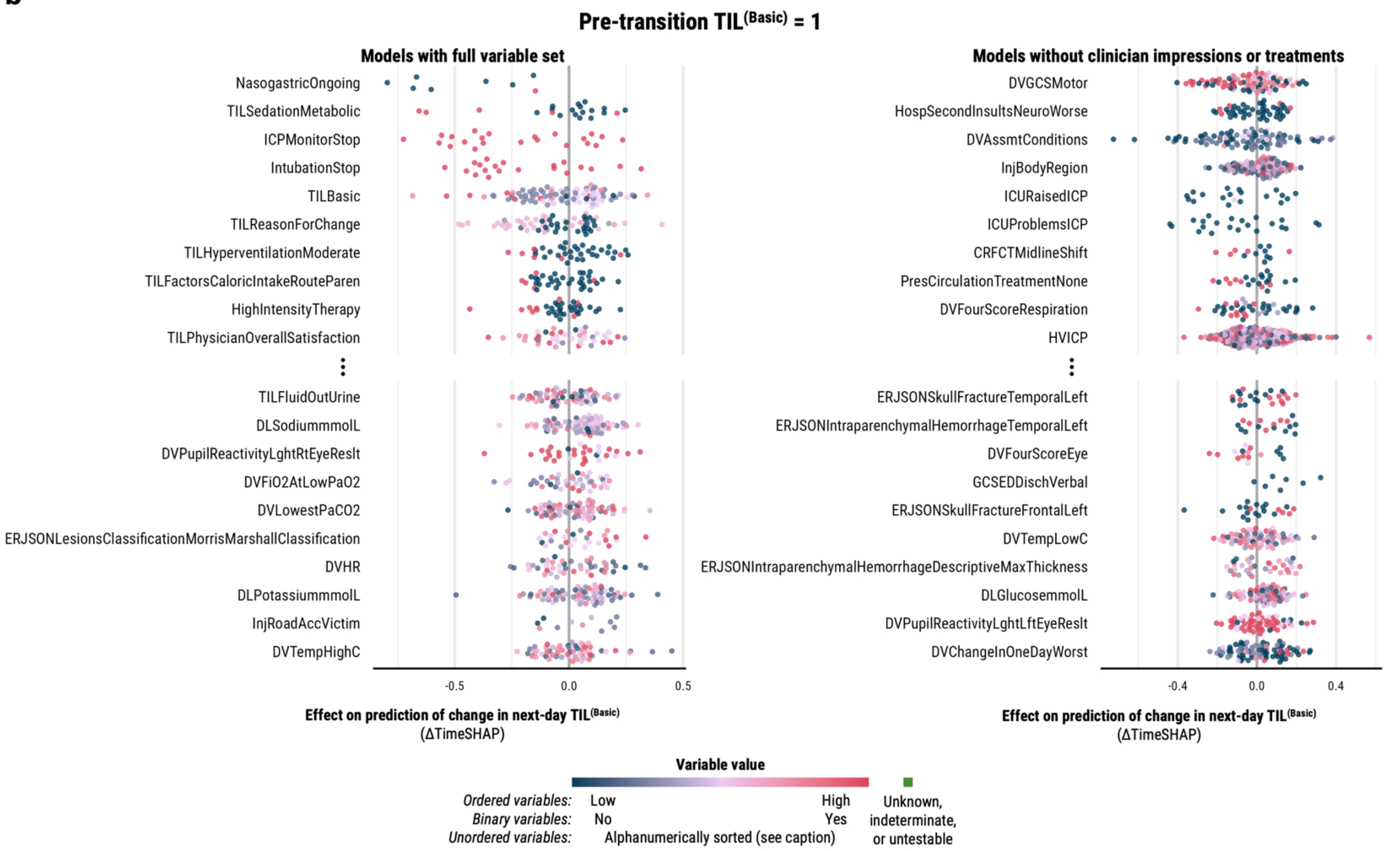

Supplementary Fig. S5 (*continued*).

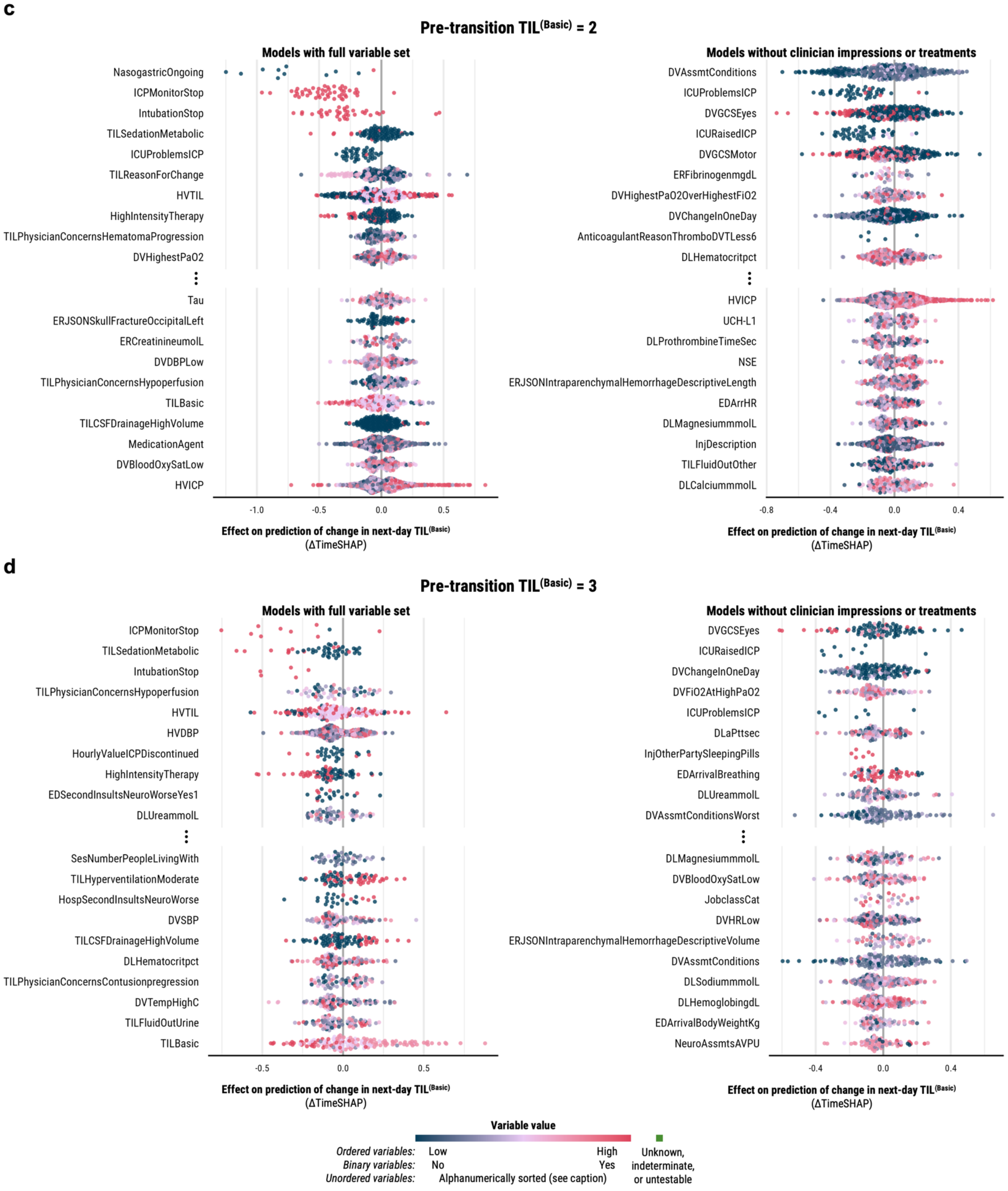

Supplementary Fig. S5 (*continued*).
e

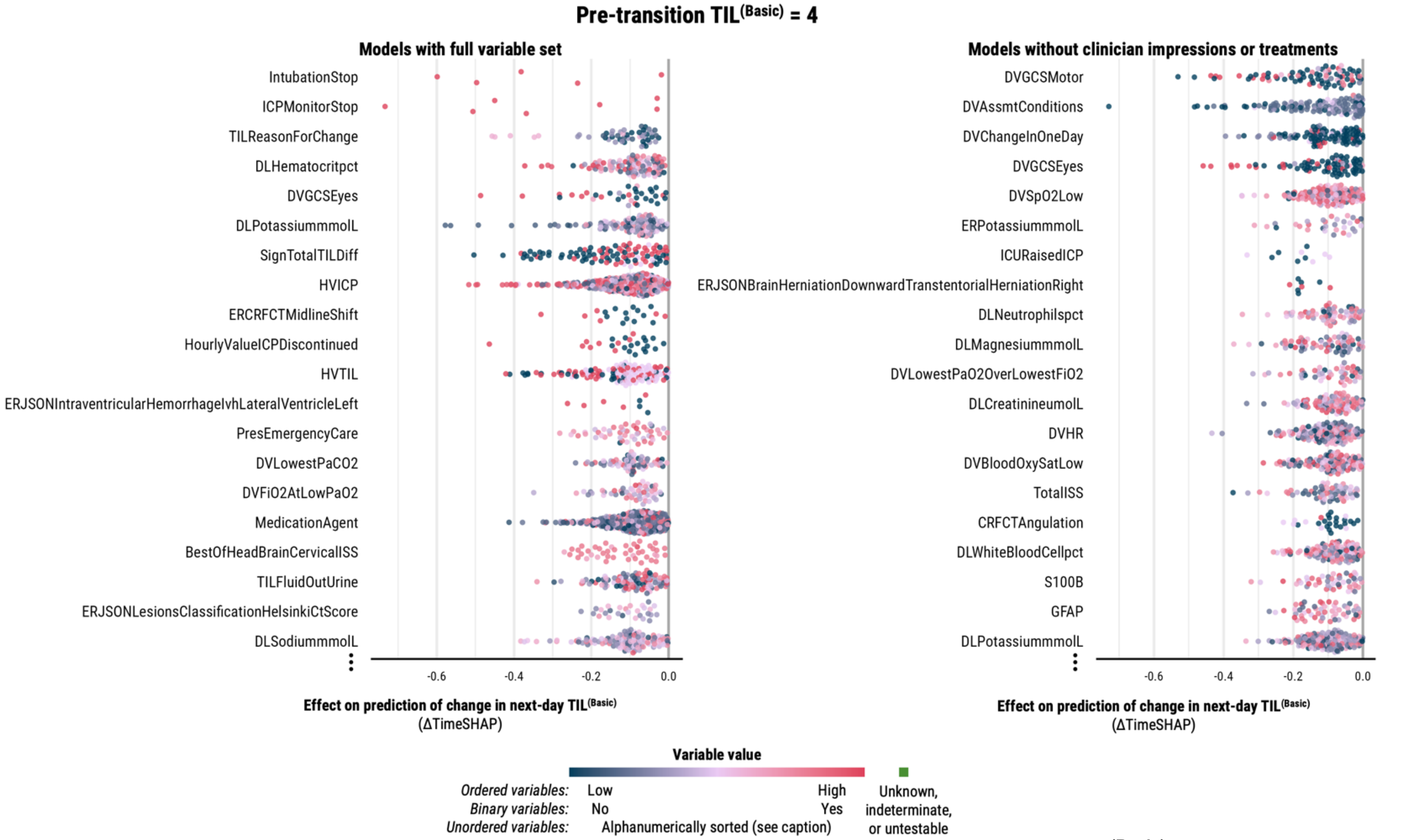

Supplementary Fig. S5. Population-level ΔTimeSHAP values stratified by pre-transition TIL$^{(Basic)}$ score. Within each panel (**a–e**), the ΔTimeSHAP values on the left-hand side are from the models trained on the full variable set whilst the ΔTimeSHAP values on the right-hand side are from the models trained without clinician impressions or treatments. ΔTimeSHAP values are interpreted as the relative contributions of variables towards the difference in model prediction of next-day TIL$^{(Basic)}$ over the two days directly preceding the change in TIL$^{(Basic)}$ (Supplementary Methods S5). Therefore, the study population represented in this figure is limited to patients who experienced a change in TIL$^{(Basic)}$ after day two of ICU stay (n=575). The variables were selected by first identifying the ten variables with non-missing value tokens with the most negative median ΔTimeSHAP values across the population (above the ellipses) and then, amongst the remaining variables, selecting the ten with non-missing value tokens with the most positive median ΔTimeSHAP values (below the ellipses). Each point represents the mean ΔTimeSHAP value, taken across all 20 repeated cross-validation partitions, for a token preceding an individual patient's change in TIL$^{(Basic)}$. The colour of the point represents the relative ordered value of a token within a variable, and for unordered variables (e.g., patient status during GCS assessment), tokens were sorted alphanumerically (the sort index per possible unordered variable token is provided in the CENTER-TBI data dictionary: https://www.center-tbi.eu/data/dictionary). All abbreviated variable names are decoded in the CENTER-TBI data dictionary.

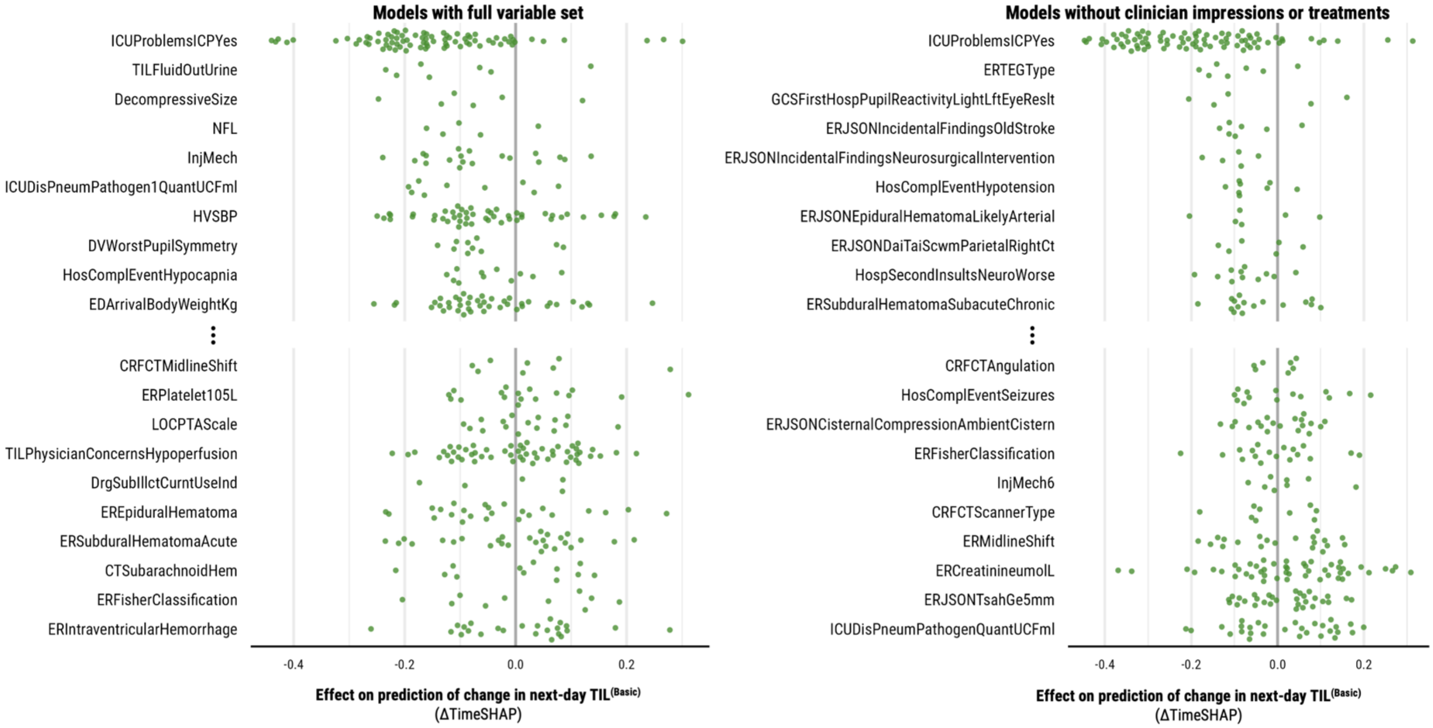

Supplementary Fig. S6. Population-level ΔTimeSHAP values for missing value tokens. The ΔTimeSHAP values on the left panel are from the models trained on the full variable set whilst the ΔTimeSHAP values on the right panel are from the models trained without clinician impressions or treatments. ΔTimeSHAP values are interpreted as the relative contributions of a variable's missingness towards the difference in model prediction of next-day $TIL^{(Basic)}$ over the two days directly preceding the change in $TIL^{(Basic)}$ (Supplementary Methods S5). Therefore, the study population represented in this figure is limited to patients who experienced a change in $TIL^{(Basic)}$ after day two of ICU stay (*n*=575). The variables were selected by first identifying the ten variables with missing value tokens with the most negative median ΔTimeSHAP values across the population (above the ellipses) and then, amongst the remaining variables, selecting the ten with missing value tokens with the most positive median ΔTimeSHAP values (below the ellipses). Each point represents the mean ΔTimeSHAP value, taken across all 20 repeated cross-validation partitions, for a token preceding an individual patient's change in $TIL^{(Basic)}$. All abbreviated variable names are decoded in the CENTER-TBI data dictionary: https://www.center-tbi.eu/data/dictionary.

SUPPLEMENTARY TABLES

NOTE: Static variables are those with values fixed at ICU admission (e.g., helmet on during accident?). Intervention variable directly represent a treatment or management decision performed during a patient's ICU stay (i.e., administration of hypertonic saline). Since an intervention variable must take place during a patient's ICU stay, a variable cannot be both a static and an intervention variable. However, a variable can be both not static (i.e., dynamic) and not an intervention (e.g., a result from an ICU lab test or imaging report).

Supplementary Table S1. Manually excluded variables indicating death or withdrawal of life-sustaining treatment.

Name	Format	Description	Possible Values
BrainDeathDate	date	Reflects the date of Brain death in case of Withdrawal of life-sustaining measures	Not Applicable
BrainDeathTime	text	Reflects the Time of Brain death in case of Withdrawal of life -sustaining measures Check also "Hospital.BrainDeathDate" for the date.	Not Applicable
CTPatientLocation	text	This variable describes the in-hospital location of the patient when the CT-scan was performed and was not meant to describe the locat ion of the CT-scanner. Three options: ER, Ward/Admission, ICU	ADMIS=Ward/Admission;ED=ER;ICU=ICU
DeadAge	integer	Reflects if the reason for Withdrawal of life-sustaining measures was age.	0=No;1=Yes;88=Unknown
DeadCoMorbidities	integer	Reflects if the reason for Withdrawal of life-sustaining measures was co-morbidities.	0=No;1=Yes;88=Unknown
DeadDeterminationOfBrainDe ath	integer	Reflects if the reason for Withdrawal of life-sustaining measures was Determination of brain death (acco rding to national law).	0=No;1=Yes;88=Unknown
DeadOrganDonation	integer	Reflects if Withdrawal of life-sustaining measures was followed by organ donation.	0=No;1=Yes;88=Unknown
DeadPatWill	integer	Reflects if the reason for Withdrawal of life-sustaining measures was Following living will of patient.	0=No;1=Yes;88=Unknown
DeadRequestRelatives	integer	Reflects if the reason for Withdrawal of life-sustaining measures was On request of relatives.	0=No;1=Yes;88=Unknown
DeadSeverityofTBI	integer	Reflects if the reason for Withdrawal of life-sustaining measures was Severity of TBI.	0=No;1=Yes;88=Unknown
DeathAutopsy	integer	Reflects if an autopsy was performed after the death of the p atient.	0=No;1=Yes, forensic;2=Yes, clinical;88=Unknown
DeathCause	integer	Cause of death in or outside the hospital	1=Head injury/initial injury;2=Head injury/secondary intracranial damage;3=Systemic trauma;4=Medical complications;88=Unknown;99=Other
DeathCauseOther	text	"Other" cause of death in or outside the hospital (than predef ined list).	Not Applicable
DeathDate	date	Date of death also recorded on hospital discharge and at foll owup: FollowUp.FUPrincipalDeathCause;Death may also ha ve been recorded in the ER forms: Subject.DeathDate	Not Applicable
DeathERDeclaredBrainDeadF ollowingNationalCriteria	integer	Reflects if patient was declared brain dead following national criteria. Only applicable if patient declared "dead" on the ER	0=No;1=Yes;88=Unknown
DeathERDOA	integer	Reflects if patient is declared dead on the ER --> Dead on arrival (DOA). Only applicable if patient declared "dead" on the ER.	0=No;1=Yes;88=Unknown
DeathERUnsuccResusForExt raCranInj	integer	Reflects unsuccessful resuscitation for extra cranial injuries if patient is declared dead on the ER. Only applicable if patient declared "dead" on the ER	0=No;1=Yes;88=Unknown
DeathERWithdrawalLifeSupp ForSeverityOfTBI	integer	Reflects Withdrawal of life¬≠sustaining measures for severity of TBI if patient is declared dead on the ER. Only applicable if patient declared "dead" on the ER	0=No;1=Yes;88=Unknown
DeathTime	text	Time of death	Not Applicable
DischargeStatus	integer	Assessment by investigator Reflects if the patient was dead or alive at discharge.	0=Dead;1=Alive;88=Unknown
EOSReason	integer	Reason for end of study participation	1=Completion of study;2=Inability to obtain follow-up;3=Withdrawal from study (by patient or represen tative);4=Adverse event(s);5=Decision for DNR*:;6=Withdrawal of support;7=Death;99=Other
EOSReasonOtherTxt	text	"Other" reason for end of study participation than the predefi ned list.	Not Applicable
ICDCode1	text	Up to 16 fields available to enter diagnosis as recorded by h ospital administration according to ICD codes; applicable to patients admitted/discharged from hospital. For patients discharged directly from the ER, ICD codes are documented in: InjuryHx.ERDestICDCodes1	Not Applicable

Name	Format	Description	Possible Values
ICDCode2	text	Up to 16 fields available to enter diagnosis as recorded by h ospital administration according to ICD codes; applicable to patients admitted/discharged from hospital.<br>For patients discharged directly from the ER, ICD codes are documented in: InjuryHx.ERDestICDCodes1	Not Applicable
ICDCode3	text	Up to 16 fields available to enter diagnosis as recorded by h ospital administration according to ICD codes; applicable to patients admitted/discharged from hospital.<br>For patients discharged directly from the ER, ICD codes are documented in: InjuryHx.ERDestICDCodes1	Not Applicable
ICDCode4	text	Up to 16 fields available to enter diagnosis as recorded by h ospital administration according to ICD codes; applicable to patients admitted/discharged from hospital.<br>For patients discharged directly from the ER, ICD codes are documented in: InjuryHx.ERDestICDCodes1	Not Applicable
ICDCode5	text	Up to 16 fields available to enter diagnosis as recorded by h ospital administration according to ICD codes; applicable to patients admitted/discharged from hospital.<br>For patients discharged directly from the ER, ICD codes are documented in: InjuryHx.ERDestICDCodes1	Not Applicable
ICDCode6	text	Up to 16 fields available to enter diagnosis as recorded by h ospital administration according to ICD codes; applicable to patients admitted/discharged from hospital.<br>For patients discharged directly from the ER, ICD codes are documented in: InjuryHx.ERDestICDCodes1	Not Applicable
ICDCodeVersion	integer	This variable reflects if the ICD code version 9 or version 10 was used.<br>Up to 16 fields are available to enter diagnosis as recorded b y hospital administration according to ICD codes.	9=ICD-9;10=ICD-10
ICPStopReason	integer	Reason for stopping ICP. Also check Hospital.ICPMonitorSto p.	1=Clinically improved;2=ICP stable and < 20 mmHg ;3=Monitor/catheter failure;4=Patient considered un salvagable;5=Patient died;99=Other
ICPStopReason	integer	Reason for stopping ICP. Also check Hospital.ICPMonitorSto p.	1=Clinically improved;2=ICP stable and < 20 mmHg ;3=Monitor/catheter failure;4=Patient considered un salvagable;5=Patient died;99=Other
ICUDischargeICDCode1	text	The intent here is to register ICD code as recorded in hospit al administrative files for patients directly discharged from th e ICU. Up to 16 codes can be entered, ICD codes are further captured at ER discharge and at hospital discharge.	Not Applicable
ICUDischargeICDCode2	text	The intent here is to register ICD code as recorded in hospit al administrative files for patients directly discharged from th e ICU. Up to 16 codes can be entered, ICD codes are further captured at ER discharge and at hospital discharge.	Not Applicable
ICUDischargeICDCode3	text	The intent here is to register ICD code as recorded in hospit al administrative files for patients directly discharged from th e ICU. Up to 16 codes can be entered, ICD codes are further captured at ER discharge and at hospital discharge.	Not Applicable
ICUDischargeICDCode4	text	The intent here is to register ICD code as recorded in hospit al administrative files for patients directly discharged from th e ICU. Up to 16 codes can be entered, ICD codes are further captured at ER discharge and at hospital discharge.	Not Applicable
ICUDischargeICDCode5	text	The intent here is to register ICD code as recorded in hospit al administrative files for patients directly discharged from th e ICU. Up to 16 codes can be entered, ICD codes are further captured at ER discharge and at hospital discharge.	Not Applicable
ICUDischargeICDCode6	text	The intent here is to register ICD code as recorded in hospit al administrative files for patients directly discharged from th e ICU. Up to 16 codes can be entered, ICD codes are further captured at ER discharge and at hospital discharge.	Not Applicable
ICUDischargeICDCodeVersio n	integer	This variable reflects if the ICD code version 9 or version 10 was used.<br>Up to 16 fields are available to enter diagnosis as recorded b y hospital administration according to ICD codes.	9=9;10=10
ICUDischargeStatus	integer	Reflects if patient was alive or dead on discharge from ICU	1=Alive;2=Dead;88=Unknown
ICUDischargeTo	integer	Reflects location to which the patient was discharged from I CU	1=General ward;2=Other ICU;3=Other hospital;4=R ehab unit;5=Home;6=Nursing home;7=Step down/h igh care unit;88=Unknown;99=Other
ICUDischargeToOther	text	Specifies the "other" location to which the patient was discha rged from ICU Check also "Hospital.ICUDischargeTo"	Not Applicable
ICUDisPatDeadAtICU	integer	Reflects if the patient was declared dead on the ICU.<br>Intended as an introductory question for the details on withdr awal of treatment, brain death and organ donation	0=No;1=Yes

Name	Format	Description	Possible Values
ICUDisSupportWithdrawnDate	date	This variable documents date and time at which life prolonging therapy was withdrawn (together with "Hospital.ICUDisSupportWithdrawnTime")	Not Applicable
ICUDisSupportWithdrawnTime	text	This variable documents date and time at which life prolonging therapy was withdrawn (together with "Hospital.ICUDisSupportWithdrawnDate")	Not Applicable
ICUDisWithdrawalTreatmentDecisionDate	date	Investigators were requested to record the details of Withdrawal of Treatment or Life support if applicable.	Not Applicable
ICUDisWithdrawalTreatmentDecisionTime	text	Investigators were requested to record the details of Withdrawal of Treatment or Life support if applicable.	Not Applicable
ICUDisWithdrawlTreatmentDecision	integer	Investigators were requested to record the details of Withdrawal of Treatment or Life support if applicable.	1=Multi disciplinary;2=By a single physician;3=With relatives
IntubationStopReason	integer	This variable describes the Stop Reason of Extubation in case of Ventilation Management (only for ICU patients).	1=Respiratory stable;2=Accidental;3=Withdrawal of care
LengthOfStay	decimal	This variable reflects the length of stay of the patient at the study hospital.<br>It has been derived using the information of the date and time of arrival at the study hospital and date and time of (study) hospital discharge.	Not Applicable
MonJugularSatStopReason	integer	Beside brain specific ICP monitoring in the ICU, details were recorded on other types of monitoring in the ICU.<br>This variable reflects the reason for stopping if there was Jugular oximetry.	1=Monitor/catheter failure;2=Patient considered unsalvageable;3=Patient died;4=Clinically no longer required
MonLicoxStopReason	integer	Beside brain specific ICP monitoring in the ICU, details were recorded on other types of monitoring in the ICU.<br>This variable reflects the reason for stopping if there was Brain tissue PO2 monitoring.	1=Monitor/catheter failure;2=Patient considered unsalvageable;3=Patient died;4=Clinically no longer required
MonLicoxStopReason	integer	Beside brain specific ICP monitoring in the ICU, details were recorded on other types of monitoring in the ICU.<br>This variable reflects the reason for stopping if there was Brain tissue PO2 monitoring.	1=Monitor/catheter failure;2=Patient considered unsalvageable;3=Patient died;4=Clinically no longer required
MonMicrodialysisStopReason	integer	Beside brain specific ICP monitoring in the ICU, details were recorded on other types of monitoring in the ICU.<br>This variable reflects the reason for stopping if there was Microdialysis.	1=Monitor/catheter failure;2=Patient considered unsalvageable;3=Patient died;4=Clinically no longer required
OrganDonationDate	date	Reflects the date of organ donation in case of Withdrawal of life-sustaining measures, if applicable.	Not Applicable
OrganDonationTime	text	Reflects the time of organ donation in case of Withdrawal of life-sustaining measures, if applicable.	Not Applicable
SupportWithdrawnDate	date	Investigators were requested to record the details of Withdrawal of Treatment or Life support if applicable.	Not Applicable
SupportWithdrawnTime	text	Investigators were requested to record the details of Withdrawal of Treatment or Life support if applicable.	Not Applicable
TimeSinceICUAdmisDeath	text	Investigators were requested to record the details of Withdrawal of Treatment or Life support if applicable.<br>This reflects the time between admission in the ICU and death	Not Applicable
WithdrawalOption	integer	In case of complete withdrawal, all data have been deleted from the database	1=Complete Withdrawal (no further contact, destruction of all data and samples collected up to that point);2=No further study related activities, but consent to access of clinical notes and use of existing data
WithdrawalTreatmentDecision	integer	Intended only to be scored if a medical decision was made to withdraw active treatment because of anticipated poor prognosis. However, some investigators may have scored this when patients had recovered to an extent that active treatment was no longer necessary.	1=Multi disciplinary;2=By a single physician;3=With relatives
WithdrawalTreatmentDecisionDate	date	Investigators were requested to record the details of Withdrawal of Treatment or Life support if applicable.<br>Intended only to be scored if a medical decision was made to withdraw active treatment because of anticipated poor prognosis. However, some investigators may have scored this when patients had recovered to an extent that active treatment was no longer necessary.	Not Applicable
WithdrawalTreatmentDecisionTime	text	Investigators were requested to record the details of Withdrawal of Treatment or Life support if applicable.<br>Intended only to be scored if a medical decision was made to withdraw active treatment because of anticipated poor prognosis. However, some investigators may have scored this when patients had recovered to an extent that active treatment was no longer necessary.	Not Applicable

Name	Format	Description	Possible Values
WithdrawSuppDateTime	datetime	Withdrawal of Support Date & Time if Withdrawal of life-sustaining support was the reason for end of study participation.	Not Applicable

Supplementary Table S2. Physician-based impression variables.

Name	Static	Format	Description	Possible Values
InjAIS	TRUE	integer	In the original AIS classification of injury severity, the grading is from 1 (minor) to 6 (unsurvivable). We added a score of 0 to designate absence of injuries. This is the AIS score for body regions as specified by AIS.InjBodyRegion.	0=None;1=Minor: no treatment needed;2=Moderate: requires only outpatient treatment;3=Serious: requires non-ICU hospital admission;4=Severe: requires ICU observation and/or basic treatment;5=Critical: requires intubation, mechanical ventilation or vasopressors for blood pressure support;6=Unsurvivable: not survivable
HVTILChangeReason	FALSE	integer	Bihourly reason for change in therapy intensity level	1=Intensified: Clinical deterioration;2=Intensified: Suspicion of increased of ICP (not measured);3=Intensified: Increased ICP (documented);4=Intensified: Clinical decision to target other mechanism;5=Intensified: Change of doctor (different shift);6=Decreasing: Clinical improvement;7=Decreasing: Adequate control over ICP;8=Decreasing: Upper treatment limit reached/past;9=Decreasing: Further treatment considered futile;10=Decreasing: Change of doctor (different shift)
TILPhysicianOverallSatisfaction	FALSE	integer	This variable aims to capture the overall satisfaction of the physician with the clinical course of this patient; "not at all satisfied" would indicate that the patient did much more poorly than expected; "very satisfied" would indicate that the patient did much better than expected. Physician satisfaction should be assessed on a daily basis,	0=Not at all;1=Slightly;2=Moderately;3=Quite;4=Very
TILPhysicianOverallSatisfactionSurvival	FALSE	integer	This variable aims to capture the opinion of the treating physician as to whether the short time survival change have chnged in comparision to the previous assessment	1=Much worse;2=A little worse;3=Unchanged;4=A little better;5=Much better
TILReasonForChange	FALSE	integer	Reflects the reason for change in TIL therapy over the day.	0=No change;1=Intensified: Clinical deterioration;2=Intensified:Suspicion of increased of ICP (not measured);3=Intensified:Increased ICP (documented);4=Intensified:Clinical decision to target other mechanism;5=Intensified:Change of doctor (different shift);6=Decreasing:Clinical improvement;7=Decreasing:Adequate control over ICP;8=Decreasing:Upper treatment limit reached/past;9=Decreasing:Further treatment considered futile;10=Decreasing:Change of doctor (different shift)
TotalTIL	FALSE	integer	Calculated centrally - 24 hour TILS as the worst sum TILs for each day for the ICU timepoints (day 1-7, 10, 14, 21 and 28)	Not Applicable
AbdomenPelvicContentsAIS	TRUE	integer	AIS score for the Abdomen/Pelvic Contents In the original AIS classification of injury severity, the grading is from 1 (minor) to 6 (unsurvivable). We added a score of 0 to designate absence of injuries.	0=None;1=Minor: no treatment needed;2=Moderate: requires only outpatient treatment;3=Serious: requires non-ICU hospital admission;4=Severe: requires ICU observation and/or basic treatment;5=Critical: requires intubation, mechanical ventilation or vasopressors for blood pressure support;6=Unsurvivable: not survivable
BaselineGOS6MoExpectedDeathRisk	TRUE	text	At ER discharge, physician estimate of six month outcome was recorded as a baseline risk assessment: "Given all current available information, what is, in your subjective opinion, the most likely 6-month outcome of this patient? To be based upon information on discharge ER or admission to hospital/ICU". This reflects the Risk of death in %	Not Applicable
BaselineGOS6MoExpectedOutcome	TRUE	text	At ER discharge, physician estimate of six month outcome was recorded as a baseline risk assessment: "Given all current available information, what is, in your subjective opinion, the most likely 6-month outcome of this patient? To be based upon information on discharge ER or admission to hospital/ICU". This reflects the Expected outcome (GOS)	D=D - Death;GR=GR - Good Recovery;MD=MD - Moderate Disability;SD=SD - Severe Disability;V=V - Vegetative State
BaselineGOS6MoUnfavourableOutcomeRisk	TRUE	text	At ER discharge, physician estimate of six month outcome was recorded as a baseline risk assessment: "Given all current available information, what is, in your subjective opinion, the most likely 6-month outcome of this patient? To be based upon information on discharge ER or admission to hospital/ICU". This reflects the Risk of unfavorable outcome (D, VS, SD) in %	Not Applicable
BaselinePhysEstOf6MoOutcomePhysicianQual	TRUE	integer	At ER discharge, physician estimate of six month outcome was recorded as a baseline risk assessment: "Given all current available information, what is, in your	1=Resident;2=Junior staff (< 5 years);3=Senior staff (>= 5 years);4=Head of department

			subjective opinion, the most likely 6-month outcome of this patient? To be based upon information on discharge ER or admission to hospital/ICU". This reflects the qualification of the physician who provided prognostic estimate on ER discharge/admission to hospital/ICU	
BaselinePhysEstOf6MoOutcomePhysicianType	TRUE	integer	At ER discharge, physician estimate of six month outcome was recorded as a baseline risk assessment: "Given all current available information, what is, in your subjective opinion, the most likely 6-month outcome of this patient? To be based upon information on discharge ER or admission to hospital/ICU". This reflects the type of the physician who provided prognostic estimate on ER discharge/admission to hospital/ICU	1=ER Physician;2=Intensive Care;3=Neurology;4=Neurosurgery;5=Traumatology;88=Unknown
BestOfAbdomenPelvicLumbarISS	TRUE	integer	AbdomenPelvicLumbar region (Highest AIS of the region)^2 compare AbdomenPelvicContentsAIS, LumbarSpineAIS. This score is taken forward for ISS calculation	Not Applicable
BestOfChestSpineISS	TRUE	integer	(highest AIS of the region)^2 Compare ThoraxChestAIS, ThoracicSpineAIS and select the highest for ISS calculation	Not Applicable
BestOfExternalISS	TRUE	integer	External region (ExternaAIS)^2 select the highest external AIS severity code for ISS calculation.	Not Applicable
BestOfExtremitiesISS	TRUE	integer	Extremities region (Highest AIS of the region)^2 compare UpperExtremitiesAIS, LowerExtremitiesAIS, PelvicGirdleAIS select the highest for ISS calculation	Not Applicable
BestOfFaceISS	TRUE	integer	Face region (FaceAIS)^2 select the highest facial injury for ISS calculation	Not Applicable
BestOfHeadBrainCervicalISS	TRUE	integer	HeadBrainCervical region (Highest AIS of the region)^2 Compare HeadNeckAIS, InjuryHx.BrainInjuryAIS, CervicalSpineAIS select the highest scoring injury in any of these 3 areas for ISS calculation	Not Applicable
BrainInjuryAIS	TRUE	integer	AIS score for the Brain Injury In the original AIS classification of injury severity, the grading is from 1 (minor) to 6 (unsurvivable). We added a score of 0 to designate absence of injuries.	0=None;1=Minor: no treatment needed;2=Moderate: requires only outpatient treatment;3=Serious: requires non-ICU hospital admission;4=Severe: requires ICU observation and/or basic treatment;5=Critical: requires intubation, mechanical ventilation or vasopressors for blood pressure support;6=Unsurvivable: not survivable
CervicalSpineAIS	TRUE	integer	AIS score for the Cervical Spine region. In the original AIS classification of injury severity, the grading is from 1 (minor) to 6 (unsurvivable). We added a score of 0 to designate absence of injuries.	0=None;1=Minor: no treatment needed;2=Moderate: requires only outpatient treatment;3=Serious: requires non-ICU hospital admission;4=Severe: requires ICU observation and/or basic treatment;5=Critical: requires intubation, mechanical ventilation or vasopressors for blood pressure support;6=Unsurvivable: not survivable
DispER	TRUE	integer	Destination of the patient at ER discharge.	1=Discharge home;2=Discharge other facility;3=Hospital admission--Ward;4=Hospital admission--Intermediate/high care unit;5=Hospital admission--ICU;6=Hospital admission--OR for immediate surgical procedure;7=Death;8=Hospital admission--Other (e.g. observation unit);88=Unknown
EmerSurgIntraCranSurviveNoSurg	TRUE	integer	"InjuryHx.EmerSurgIntraCranSurviveNoSurg" and "InjuryHx.EmerSurgIntraCranSurviveYesSurg" These 2 variables aim to capture information on the surgeon’s expectations, eg if the surgeon considers a realistic expectation of benefit, or performs the surgery as a "last resort" in a likely hopeless case. ‘The short term survival chances of the patients if I DO NOT operate will be (in %)’	Not Applicable
EmerSurgIntraCranSurviveYesSurg	TRUE	integer	"InjuryHx.EmerSurgIntraCranSurviveNoSurg" and "InjuryHx.EmerSurgIntraCranSurviveYesSurg" These 2 variables aim to capture information on the surgeon’s expectations, eg if the surgeon considers a realistic expectation of benefit, or performs the surgery as a "last resort" in a likely hopeless case. ‘The short term survival chances of the patients if I DO operate will be (in %)’	Not Applicable
ExternaAIS	TRUE	integer	AIS score for the External skin In the original AIS classification of injury severity, the grading is from 1 (minor) to 6 (unsurvivable). We added a score of 0 to designate absence of injuries.	0=None;1=Minor: no treatment needed;2=Moderate: requires only outpatient treatment;3=Serious: requires non-ICU hospital admission;4=Severe: requires ICU observation and/or basic treatment;5=Critical: requires intubation, mechanical ventilation or vasopressors for blood pressure support;6=Unsurvivable: not survivable
FaceAIS	TRUE	integer	AIS score for Face (incl.maxillofacial) In the original AIS classification of injury severity, the grading is from 1 (minor) to 6 (unsurvivable). We added a score of 0 to designate absence of injuries.	0=None;1=Minor: no treatment needed;2=Moderate: requires only outpatient treatment;3=Serious: requires non-ICU hospital admission;4=Severe: requires ICU observation and/or basic treatment;5=Critical: requires

				intubation, mechanical ventilation or vasopressors for blood pressure support;6=Unsurvivable: not survivable
HeadNeckAIS	TRUE	integer	AIS score for the Head Neck region In the original AIS classification of injury severity, the grading is from 1 (minor) to 6 (unsurvivable). We added a score of 0 to designate absence of injuries.	0=None;1=Minor: no treatment needed;2=Moderate: requires only outpatient treatment;3=Serious: requires non-ICU hospital admission;4=Severe: requires ICU observation and/or basic treatment;5=Critical: requires intubation, mechanical ventilation or vasopressors for blood pressure support;6=Unsurvivable: not survivable
LowerExtremitiesAIS	TRUE	integer	AIS score for the Lower extremities. In the original AIS classification of injury severity, the grading is from 1 (minor) to 6 (unsurvivable). We added a score of 0 to designate absence of injuries.	0=None;1=Minor: no treatment needed;2=Moderate: requires only outpatient treatment;3=Serious: requires non-ICU hospital admission;4=Severe: requires ICU observation and/or basic treatment;5=Critical: requires intubation, mechanical ventilation or vasopressors for blood pressure support;6=Unsurvivable: not survivable
LumbarSpineAIS	TRUE	integer	AIS score for the Lumbar Spine region. In the original AIS classification of injury severity, the grading is from 1 (minor) to 6 (unsurvivable). We added a score of 0 to designate absence of injuries.	0=None;1=Minor: no treatment needed;2=Moderate: requires only outpatient treatment;3=Serious: requires non-ICU hospital admission;4=Severe: requires ICU observation and/or basic treatment;5=Critical: requires intubation, mechanical ventilation or vasopressors for blood pressure support;6=Unsurvivable: not survivable
PelvicGirdleAIS	TRUE	integer	AIS score for the Pelvic Girdle region. In the original AIS classification of injury severity, the grading is from 1 (minor) to 6 (unsurvivable). We added a score of 0 to designate absence of injuries.	0=None;1=Minor: no treatment needed;2=Moderate: requires only outpatient treatment;3=Serious: requires non-ICU hospital admission;4=Severe: requires ICU observation and/or basic treatment;5=Critical: requires intubation, mechanical ventilation or vasopressors for blood pressure support;6=Unsurvivable: not survivable
SurgIntervenAppro	TRUE	integer	WHY question: How strongly does the surgeon feels that this surgical intervention is appropriate in terms of the expected benefit to final clinical outcome?	0=0;1=1;2=2;3=3;4=4;5=5;6=6;7=7;8=8;9=9;10=10
ThoracicSpineAIS	TRUE	integer	AIS score for the Thoracic spine Region In the original AIS classification of injury severity, the grading is from 1 (minor) to 6 (unsurvivable). We added a score of 0 to designate absence of injuries.	0=None;1=Minor: no treatment needed;2=Moderate: requires only outpatient treatment;3=Serious: requires non-ICU hospital admission;4=Severe: requires ICU observation and/or basic treatment;5=Critical: requires intubation, mechanical ventilation or vasopressors for blood pressure support;6=Unsurvivable: not survivable
ThoraxChestAIS	TRUE	integer	AIS score for the Thorax Chest region. In the original AIS classification of injury severity, the grading is from 1 (minor) to 6 (unsurvivable). We added a score of 0 to designate absence of injuries.	0=None;1=Minor: no treatment needed;2=Moderate: requires only outpatient treatment;3=Serious: requires non-ICU hospital admission;4=Severe: requires ICU observation and/or basic treatment;5=Critical: requires intubation, mechanical ventilation or vasopressors for blood pressure support;6=Unsurvivable: not survivable
TotalISS	TRUE	integer	The Injury Severity Score is calculated as the sum of the squares of the the 3 body regions with the highest AIS score. The max score for the ISS = 75. If any body region AIS is assigned a score of "6", the ISS is automatically set to 75 (highest score). In the calculation of the ISS, only the 6 main body regions are taken into consideration.	Not Applicable
UpperExtremitiesAIS	TRUE	integer	AIS score for the Upper extremities. In the original AIS classification of injury severity, the grading is from 1 (minor) to 6 (unsurvivable). We added a score of 0 to designate absence of injuries.	0=None;1=Minor: no treatment needed;2=Moderate: requires only outpatient treatment;3=Serious: requires non-ICU hospital admission;4=Severe: requires ICU observation and/or basic treatment;5=Critical: requires intubation, mechanical ventilation or vasopressors for blood pressure support;6=Unsurvivable: not survivable
CRFCTReason	FALSE	text	This variable contains the main reason why a CT-scan, during hospital stay, was performed. One of following options: standard follow-up, post-operative control, clinical deterioration, (suspicion of) increasing ICP, lack of improvement, unknown, other (specified in CTMRI.CTReasonOther) The reason for making an early CT-scan/ER scan can be found in: CTMRI.CTERReason	CD=Clinical deterioration;ICUADM88=Unknown;ICUADM99=Other;IICP=(Suspicion of) Increasing ICP;LOP=Lack of improvement;POC=Post-operative control;SFU=Standard follow-up
CTNoOpMotiv	FALSE	integer	WHY question: documents reason for not having an indication for (intra)cranial surgery.	0=No surgical lesion;1=Lesion present, but Acceptable/good neurologic condition;2=Lesion present, but Guideline adherence;3=Lesion present, but Little/no mass effect;4=Lesion present, but Not hospital policy;5=Lesion present, but Extremely poor prognosis;6=Lesion present, but Brain Death;7=Lesion present, but Old age;8=Lesion present, but Wish family;88=Unknown;99=Lesion present, but Other
CTNoOpMotivOther	FALSE	text	Specification, only applicable if "CTMRI.CTNoOpMotiv" was "other"	Not Applicable
CTYesOpMotiv	FALSE	integer	WHY question: documents reason for having an indication for (intra)cranial surgery.	1=Emergency/life saving;2=Clinical deterioration;3=Mass effect on CT;4=Radiological progression;5=(Suspicion

			of) raised ICP;6=Guideline adherence;7=To prevent deterioration;8=Depressed skull fracture;99=Other
CTYesOpMotivOther	FALSE	text	Free text if "CTMRI.CTYesOpMotiv" was marked as 'Other'. Relates to the WHY question: documents reason for having an indication for (intra)cranial surgery.	Not Applicable
ERCTNoOpMotiv	TRUE	integer	In emergency room: WHY question: documents reason for not having an indication for (intra)cranial surgery.	0=No surgical lesion;1=Lesion present, but Acceptable/good neurologic condition;2=Lesion present, but Guideline adherence;3=Lesion present, but Little/no mass effect;4=Lesion present, but Not hospital policy;5=Lesion present, but Extremely poor prognosis;6=Lesion present, but Brain Death;7=Lesion present, but Old age;8=Lesion present, but Wish family;88=Unknown;99=Lesion present, but Other
ERCTNoOpMotivOther	TRUE	text	In emergency room: Specification, only applicable if "CTMRI.CTNoOpMotiv" was "other"	Not Applicable
ERCTYesOpMotiv	TRUE	integer	In emergency room: WHY question: documents reason for having an indication for (intra)cranial surgery.	1=Emergency/life saving;2=Clinical deterioration;3=Mass effect on CT;4=Radiological progression;5=(Suspicion of) raised ICP;6=Guideline adherence;7=To prevent deterioration;8=Depressed skull fracture;99=Other
ERCTYesOpMotivOther	TRUE	text	In emergency room: Free text if "CTMRI.CTYesOpMotiv" was marked as 'Other'. Relates to the WHY question: documents reason for having an indication for (intra)cranial surgery.	Not Applicable
ShortTermSurvivalNoSurg	FALSE	integer	Aims to capture information on the surgeon's expectations, eg if the surgeon considers a realistic expectation of benefit, or performs the surgery as a "last resort" in a likely hopeless case --> The short term survival chances of the patient if I DO NOT operate (1-100)	Not Applicable
ShortTermSurvivalYesSurg	FALSE	integer	Aims to capture information on the surgeon's expectations, eg if the surgeon considers a realistic expectation of benefit, or performs the surgery as a "last resort" in a likely hopeless case --> The short term survival chances of the patient if I DO operate (1-100)	Not Applicable
SurgeryCranialReason	FALSE	integer	WHY Question: aims to document the reason for intracranial surgery	1=Emergency/Life saving;2=Clinical deterioration;3=Mass effect on CT;4=Radiological progression;5=suspicion of) raised ICP;6=Guideline adherence;7=To prevent deterioration
SurgeryExtraCranialReason	FALSE	integer	The extra-cranial surgeries information was to be entered in the e-CRF in tables for which you could add as many rows as you wish. The tables consisted of - Surgery start date - Surgery start time - Surgery end date - Surgery end time - Extracranial surgery code - Reason - Delay - Short time survival if you do not operate - Short time survival if you do operate	1=Emergency/Lifesaving;2=Elective;3=Treatment of complication;4=Airway management;99=Other
TransReason	FALSE	integer	WHY question: documents reason for transition of care	1=Mechanical ventilation;2=Frequent neurological observations;3=Haemodynamic invasive monitoring;4=Extracranial injuries;5=Neurological operation;6=Clinical deterioration;7=CT abnormalities;8=Clinical observation for TBI;9=No ICU bed available;10=Could be discharged home, but no adequate supervision;11=Improvement;12=Neurological deterioration;13=Systemic compilation;14=CT progression;15=Planned surgery;16=Condition stable;17=(acute) Treatment goals accomplished;18=Need to free a bed;19=Further improvement;20=Clinical rehab completed;21=Lack of improvement;22=Late neurological deterioration;23=Problems unrelated to trauma;24=Post operative care;25=Neurological complication;99=Other

SUPPLEMENTARY METHODS

Supplementary Methods S1. Description of model endpoints and outputs for TILTomorrow.

Let $\mathbf{y}^{(i)}$ represent the vector of next-day TIL$^{\text{(Basic)}}$ scores for a patient, represented by index $i \in \{1, 2, \dots, N\}$, in an assessment population of N patients:

$$\mathbf{y}^{(i)} = \left[y_1^{(i)}, y_2^{(i)}, \dots, y_{\mathcal{T}^{(i)}}^{(i)}\right]^{\mathrm{T}}$$

where $y_t^{(i)} \in \{0, 1, 2, 3, 4\}$ is the next-day TIL$^{\text{(Basic)}}$ score (Table 1) at day $t \in \{1, 2, \dots, \mathcal{T}^{(i)}\}$. In other words, $y_t^{(i)}$ is the TIL$^{\text{(Basic)}}$ score at day $t+1$, and $\mathcal{T}^{(i)} + 1$ is the number of calendar days patient i was in the ICU. In the CENTER-TBI study, $y_t^{(i)}$ was regularly recorded for $t \in \{1, 2, 3, 4, 5, 6, 9, 13, 20, 27\} \cap \{1,2, \dots, \mathcal{T}^{(i)}\}$. The softmax output layer of the TILTomorrow models returns a trajectory of estimated probabilities $\left(p_{k,t}^{(i)}\right)$ for each possible score $(k \in \{0,1,2,3,4\})$ of next-day TIL$^{\text{(Basic)}}$:

$$p_{k,t}^{(i)} = \widehat{\Pr}\left(y_t^{(i)} = k\right).$$

From score-specific probability scores, we calculated two interpretable probability scores. The first was an estimated probability at each possible threshold $\left(p_{>k,t}^{(i)}\right)$ of next-day TIL$^{\text{(Basic)}}$:

$$p_{>k,t}^{(i)} = \sum_{k'=k+1}^{4} p_{k',t}^{(i)}$$

$\forall k \in \{0, 1, 2, 3\}$. The second was the probability of TIL$^{\text{(Basic)}}$ decreasing $\left(\pi_{-1,t}^{(i)}\right)$, staying the same $\left(\pi_{0,t}^{(i)}\right)$, or increasing $\left(\pi_{1,t}^{(i)}\right)$ tomorrow in relation to the last available TIL$^{\text{(Basic)}}$ score. Let $y_0^{(i)}$ represent the TIL$^{\text{(Basic)}}$ score of the first calendar day of a patient's ICU stay. Moreover, if $y_{t-1}^{(i)}$ (i.e., today's TIL$^{\text{(Basic)}}$ score) is missing, let it be replaced with the last available TIL$^{\text{(Basic)}}$ score for the following formulae. Then, $\pi_{-1,t}^{(i)}$ is defined as:

$$\pi_{-1,t}^{(i)} = \begin{cases} 0 & \text{if } y_{t-1}^{(i)} = 0, \\ \displaystyle\sum_{k'=0}^{y_{t-1}^{(i)}-1} p_{k',t}^{(i)} & \text{otherwise.} \end{cases}$$

$\pi_{0,t}^{(i)}$ is defined as:

$$\pi_{0,t}^{(i)} = p_{y_{t-1}^{(i)},t}^{(i)}.$$

$\pi_{1,t}^{(i)}$ is defined as:

$$\pi_{1,t}^{(i)} = \begin{cases} 0 & \text{if } y_{t-1}^{(i)} = 4, \\ \displaystyle\sum_{k'=y_{t-1}^{(i)}+1}^{4} p_{k',t}^{(i)} & \text{otherwise.} \end{cases}$$

Moreover, let $\gamma_t^{(i)} \in \{-1, 0, 1\}$ be the corresponding endpoint label that represents whether the next-day TIL$^{\text{(Basic)}}$ score is a decrease, stasis, or increase from the last available TIL$^{\text{(Basic)}}$ score:

$$\gamma_t^{(i)} = \begin{cases} -1 & \text{if } y_{t-1}^{(i)} > y_t^{(i)}, \\ 0 & \text{if } y_{t-1}^{(i)} = y_t^{(i)}, \\ 1 & \text{if } y_{t-1}^{(i)} < y_t^{(i)}, \end{cases}$$

and:

$$\boldsymbol{\gamma}^{(i)} = \left[\gamma_1^{(i)}, \gamma_2^{(i)}, \dots, \gamma_{\mathcal{T}^{(i)}}^{(i)}\right]^{\mathrm{T}}.$$

Post-processing calibration

Once model weights were trained, we used vector scaling to improve the calibration (i.e., reliability) of estimated probability scores based on the validation sets. Post-processing calibration methods, including vector scaling, are described in greater detail by Guo *et al*.[R1]

The motivation behind vector scaling is to find a single linear transformation of uncalibrated logits which helps account for the effect of over-fitting on the training set. Let $\mathbf{q}_t^{(i)}$ represent the 5×1 vector of uncalibrated logits for patient i at day t, and let σ_{SM} represent the softmax function:

$$p_{k,t}^{(i)} = \sigma_{\text{SM}}\left(\mathbf{W}_t \mathbf{q}_t^{(i)} + \mathbf{b}_t\right)$$

where $\mathbf{W}_t \in \mathbb{R}^{5\times5}$ is fixed as a diagonal matrix and $\mathbf{b}_t \in \mathbb{R}^{5\times1}$. $\mathbf{W}_t$ and $\mathbf{b}_t$ are learned by training a multinomial logistic regression model between uncalibrated logits and next-day TIL$^{(\text{Basic})}$ scores on the validation set at each assessment day t.

Supplementary Methods S2. Repeated Bootstrap Bias Corrected with Dropping Cross-Validation (BBCD-CV).

To make the tuning and assessment of our hyperparametric modelling strategy computationally tractable, we implemented a slightly modified version of the Repeated Bootstrap Bias Corrected with Dropping Cross-Validation (BBCD-CV) method proposed by Tsamardinos *et al.*[R2] This method has been reported to achieve similar bias performance to nested CV with considerably greater efficiency.[R2,3]

Dropout of low-performing hyperparametric configurations

One of the challenges in training our modelling strategy is the high number of hyperparameter combinations (i.e., configurations). The intuition behind BBCD-CV is to dropout significantly low-performing configurations, determined by bias-corrected bootstrapping of validation set performance, at certain checkpoints of the repeated CV process to make training more efficient.

Let $\Theta = \{\boldsymbol{\theta}_1, \boldsymbol{\theta}_2, \ldots, \boldsymbol{\theta}_C\}$ denote the set of configurations. After training models of each of the C configurations on the training sets of the first full repeat (i.e., first five partitions), we collected all the validation set model outputs (i.e., predictions). On these outputs, we calculated two performance metrics for each of the configurations: the ordinal c-index[R4] (ORC) for discrimination as well as the macro-averaged calibration slope[R5] ($\overline{\beta_1}$) for calibration. For each metric, we selected the configuration with the optimal performance:

$$i^*_{\text{ORC}} = \arg\max_i \text{ORC}\,(\boldsymbol{\theta}_i)$$
$$i^*_{\overline{\beta_1}} = \arg\min_i \left|1 - \overline{\beta_1}(\boldsymbol{\theta}_i)\right|$$

Then, we drew 1,000 resamples of unique patients from the validation set outputs for bootstrapping and calculate the ORC and $\overline{\beta_1}$ values of each $\boldsymbol{\theta}$ in each resample. For each $\boldsymbol{\theta}_i$, we calculated the proportion of resamples in which $\boldsymbol{\theta}_i$ had a lower ORC than that of $\boldsymbol{\theta}_{i^*_{\text{ORC}}}$ as well as the proportion of resamples in which $\boldsymbol{\theta}_i$ had a higher calibration slope error $\left(\left|1 - \overline{\beta_1}(\boldsymbol{\theta}_i)\right|\right)$ than that of $\boldsymbol{\theta}_{i^*_{\overline{\beta_1}}}$. Moreover, we estimated a 95% confidence interval (CI) of $\overline{\beta_1}$ for each $\boldsymbol{\theta}_i$ based on the configuration's 2.5th and 97.5th percentile of $\overline{\beta_1}$ values across the resamples. If a configuration's proportion of lower-performing resamples was greater than 0.99 for either metric and its 95% CI of $\overline{\beta_1}$ did not include 1, then that configuration was dropped from further training or assessment.

We repeated this process after each full repeat (i.e., every five partitions), until 20 or fewer configurations remained. After training was complete on all 100 repeated CV partitions, we repeated the dropout procedure one last time to remove configurations from testing set assessment.

Confidence intervals for testing set performance

After model training and configuration dropout was complete, we assessed the performance of our modelling strategies with bias-corrected bootstrapping. We compiled the set of testing set outputs for the remaining configurations and drew 1,000 resamples of unique patients in the population for bootstrapping. We iterated through each of the resamples and determined the optimal configuration for each performance metric in the current resample. Then, we calculated the corresponding performance metric for the optimal configuration in the set of patients not in the current resample. The collection of 1,000 out-of-sample performance metric values formed the estimated distribution of the metric for statistical inference, from which the 2.5th and 97.5th percentiles formed the bounds for the metric's 95% confidence interval.

It is important to note that repeated CV assesses the performance of a modelling strategy and not the performance of a specific trained model or a specific hyperparametric configuration. The modelling strategy encompasses the full range of tested configurations, and the optimal configuration for a given metric may differ between resamples. Moreover, by choosing the optimal configuration within one set of patients and then assessing its performance in another for each resample, the BBCD-CV algorithm reduces the bias in configuration selection without needing to train additional models.

Supplementary Methods S3. Hyperparameter optimisation report.

Summary

Combinations of the listed hyperparameters were tested on the validation sets of our repeated *k*-fold cross-validation (20 repeats, 5 folds) in successive model versions. A single combination of model hyperparameters is known as a configuration. Configurations which significantly ($\alpha = 0.01$) underperformed in calibration and discrimination on the validation set were dropped out after each repeat using the Bootstrap Bias Corrected with Dropping Cross-Validation (BBCD-CV) method, as detailed in Supplementary Methods S2. For greater detail regarding the role of each hyperparameter in model function, please see the model code in https://github.com/sbhattacharyay/TILTomorrow/blob/main/scripts/models/dynamic_TTM.py. Moreover the selection of hyperparameters in this study was informed by the optimal configurations of our prior, dynamic GOSE modelling study.[R6]

Overview of tested hyperparameters

- Embedding vector dimension: length of vectors learned for each token in the embedding layer.
  - Tested values: 128, 256, 512, 1024
  - Optimal value: 512
- Recurrent neural network (RNN) architecture: type of RNN structure.
  - Tested values: long short-term memory (LSTM), gated recurrent unit (GRU)
  - Optimal value: GRU
- RNN hidden state dimension: dimension of the RNN hidden state.
  - Tested values: 128, 256, 512
  - Optimal value: 256
- Window limit during training: limit to the number of time windows per training set patient considered during training.
  - Tested values: None, 6, 13
  - Optimal value: 13
- Minimum variable representation: minimum proportion of patients with non-missing value for a variable for it to be included in the model embedding layer dictionary.
  - Tested values: None, 0.05
  - Optimal value: None
- Maximum number of tokens: maximum number of tokens a single variable can have for it to be included in the embedding layer dictionary.
  - Tested values: None, 100
  - Optimal value: None

Tested hyperparameters per model version

We had two iterations of model development. Attached are the high-dimensional parallel plots (HiPlots) to visualise the effect of hyperparameters on the validation set ordinal c-index (ORC) and calibration slope error $\left(\left|1-\overline{\beta_1}\right|\right)$.

Version 1-0:

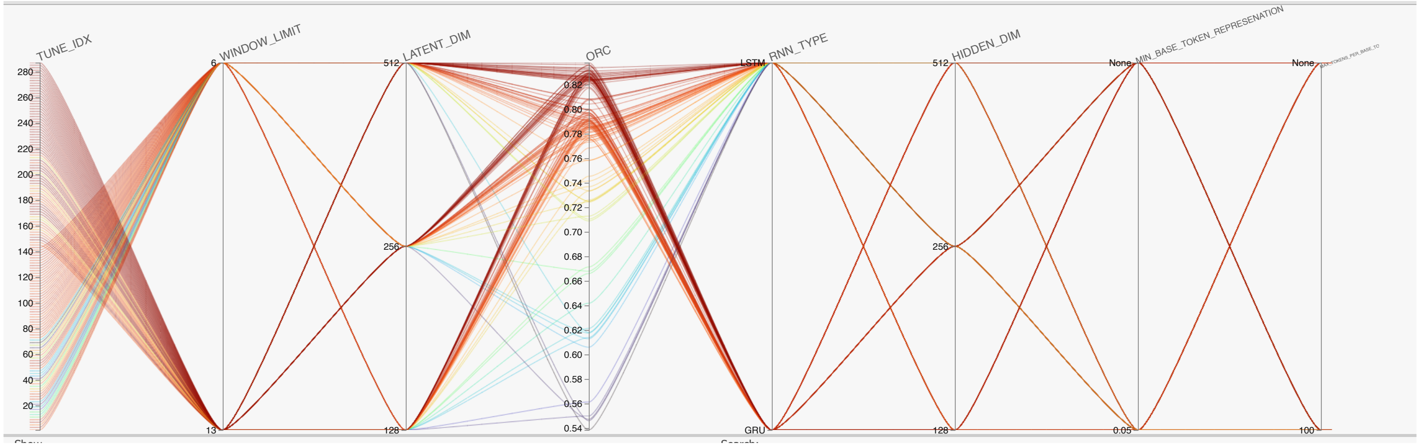

Version 1-0 HiPlots of ORC. An interactive version of the HiPlot is available on GitHub: https://sbhattacharyay.github.io/TILTomorrow/TILTomorrow_model_performance/v1-0/ORC_hiplot.html.

TUNE_IDX	WINDOW_LIMIT	RNN_TYPE	LATENT_DIM	HIDDEN_DIM	MIN_BASE_TOKEN_REPRESENATION	MAX_TOKENS_PER_BASE_TOKEN	ORC	uid	from_uid
277	13	GRU	512	256	0.05	None	0.8379508363195278	0	null
265	13	GRU	512	128	None	None	0.8362549909194259	1	null
245	13	GRU	256	128	0.05	None	0.8357516317744974	2	null
259	13	GRU	256	512	None	100	0.8355192502123274	3	null
207	13	LSTM	512	256	0.05	100	0.833763214970582	4	null
279	13	GRU	512	256	0.05	100	0.8332464485253526	5	null
283	13	GRU	512	512	None	100	0.8322209465823152	6	null
253	13	GRU	256	256	0.05	None	0.8317453677058259	7	null
243	13	GRU	256	128	None	100	0.8316034788036919	8	null
249	13	GRU	256	256	None	None	0.8313614775903667	9	null
211	13	LSTM	512	512	None	100	0.8309341709083858	10	null
285	13	GRU	512	512	0.05	None	0.8304756587307744	11	null
227	13	GRU	128	256	None	100	0.8296171243455466	12	null
261	13	GRU	256	512	0.05	None	0.8292864544753716	13	null
171	13	LSTM	256	128	None	100	0.8292560103508316	14	null
273	13	GRU	512	256	None	None	0.8290438050713731	15	null
237	13	GRU	128	512	0.05	None	0.8287387921356838	16	null
233	13	GRU	128	512	None	None	0.8285117441289452	17	null
149	13	LSTM	128	128	0.05	None	0.8284087633698562	18	null
203	13	LSTM	512	256	None	100	0.8276074143996436	19	null
247	13	GRU	256	128	0.05	100	0.8274887803012904	20	null
251	13	GRU	256	256	None	100	0.8270750403167227	21	null
287	13	GRU	512	512	0.05	100	0.8268932274408687	22	null
195	13	LSTM	512	128	None	100	0.826596759454209	23	null
271	13	GRU	512	128	0.05	100	0.8264529090181327	24	null

Version 1-0 top 25 hyperparametric configurations based on ORC. An interactive version of this chart is available on GitHub: https://sbhattacharyay.github.io/TILTomorrow/TILTomorrow_model_performance/v1-0/ORC_hiplot.html.

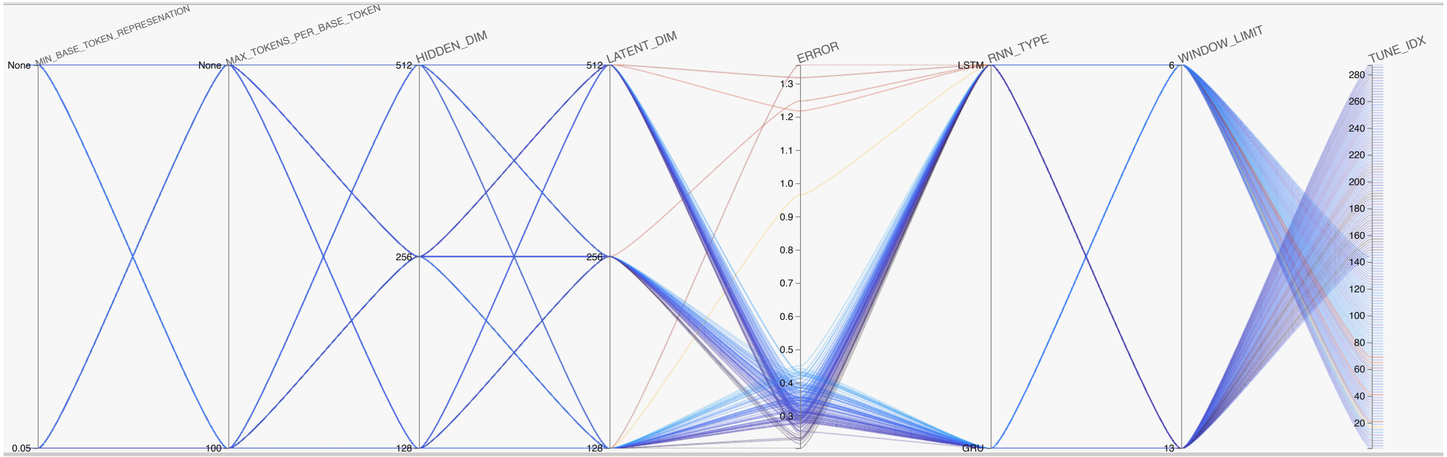

Version 1-0 HiPlots of macro-averaged calibration slope error. An interactive version of the HiPlot is available on GitHub: https://sbhattacharyay.github.io/TILTomorrow/TILTomorrow_model_performance/v1-0/thresh_calibration_hiplot.html.

TUNE_IDX	WINDOW_LIMIT	RNN_TYPE	LATENT_DIM	HIDDEN_DIM	MIN_BASE_TOKEN_REPRESENATION	MAX_TOKENS_PER_BASE_TOKEN	ERROR
157	13	LSTM	128	256	0.05	None	0.2029465656318122
191	13	LSTM	256	512	0.05	100	0.21724159025475107
171	13	LSTM	256	128	None	100	0.2264563378255279
155	13	LSTM	128	256	None	100	0.23001757761345892
187	13	LSTM	256	512	None	100	0.23400563938119193
189	13	LSTM	256	512	0.05	None	0.2341144599076415
149	13	LSTM	128	128	0.05	None	0.2349617742179136
175	13	LSTM	256	128	0.05	100	0.24222775043586983
61	6	LSTM	512	256	0.05	None	0.24819935079851194
277	13	GRU	512	256	0.05	None	0.25415229742201695
207	13	LSTM	512	256	0.05	100	0.25565114547886836
209	13	LSTM	512	512	None	None	0.2565276979970379
211	13	LSTM	512	512	None	100	0.2652089646139199
183	13	LSTM	256	256	0.05	100	0.26772146475502395
169	13	LSTM	256	128	None	None	0.2714102630505819
199	13	LSTM	512	128	0.05	100	0.2756491254201819
7	6	LSTM	128	128	0.05	100	0.2774463304798968
167	13	LSTM	128	512	0.05	100	0.27757104033660013
165	13	LSTM	128	512	0.05	None	0.27835888742558573
245	13	GRU	256	128	0.05	None	0.28036300312348
227	13	GRU	128	256	None	100	0.2830082783141855
285	13	GRU	512	512	0.05	None	0.2833660281970304
263	13	GRU	256	512	0.05	100	0.28423038020215674
213	13	LSTM	512	512	0.05	None	0.2857221826161927
261	13	GRU	256	512	0.05	None	0.2882236051565642

Version 1-0 top 25 hyperparametric configurations based on macro-averaged calibration slope error. An interactive version of this chart is available on GitHub: https://sbhattacharyay.github.io/TILTomorrow/TILTomorrow_model_performance/v1-0/thresh_calibration_hiplot.html.

Version 2-0:

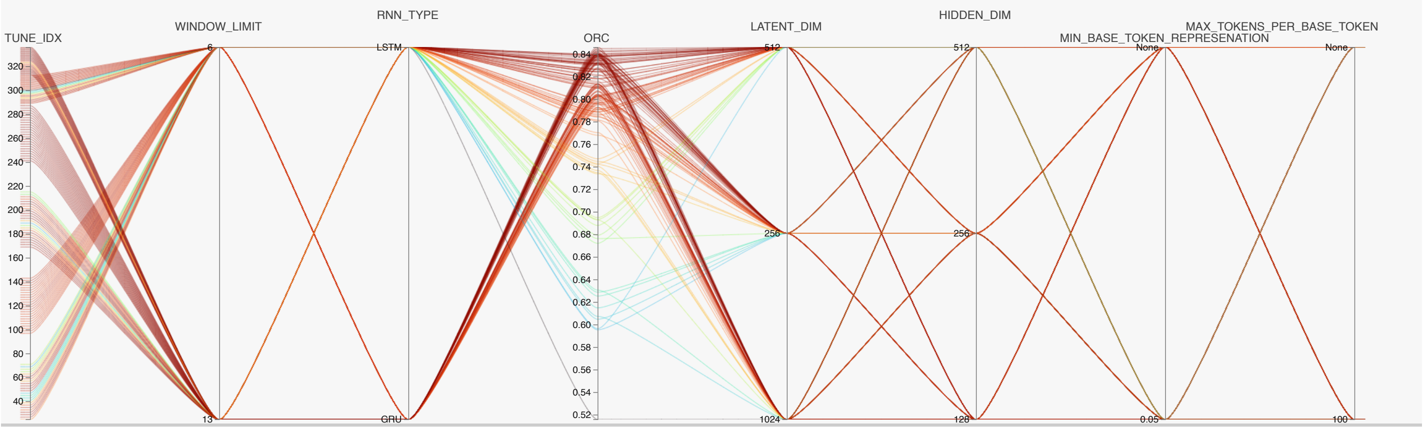

Version 2-0 HiPlots of ORC. An interactive version of the HiPlot is available on GitHub: https://sbhattacharyay.github.io/TILTomorrow/TILTomorrow_model_performance/v2-0/ORC_hiplot.html.

	TUNE_IDX	WINDOW_LIMIT	RNN_TYPE	LATENT_DIM	HIDDEN_DIM	MIN_BASE_TOKEN_REPRESENATION	MAX_TOKENS_PER_BASE_TOKEN	ORC
■	333	13	GRU	1024	512	None	None	0.8460361262840276
■	269	13	GRU	512	128	0.05	None	0.844614332489145
■	336	13	GRU	1024	512	0.05	100	0.8441096763652861
■	241	13	GRU	256	128	None	None	0.8428358220706831
■	265	13	GRU	512	128	None	None	0.8426742510973863
■	281	13	GRU	512	512	None	None	0.84168484650292
■	330	13	GRU	1024	256	None	100	0.8414519746724569
■	325	13	GRU	1024	128	None	None	0.8408002699568409
■	332	13	GRU	1024	256	0.05	100	0.8402506566109267
■	247	13	GRU	256	128	0.05	100	0.8399068920069712
■	283	13	GRU	512	512	None	100	0.8398277297507862
■	287	13	GRU	512	512	0.05	100	0.8397874373011506
■	326	13	GRU	1024	128	None	100	0.839512964715698
■	316	13	LSTM	1024	128	0.05	100	0.8394968128621728
■	251	13	GRU	256	256	None	100	0.8394796453697052
■	175	13	LSTM	256	128	0.05	100	0.8393192154613309
■	329	13	GRU	1024	256	None	None	0.8390900349902727
■	328	13	GRU	1024	128	0.05	100	0.839054413867727
■	263	13	GRU	256	512	0.05	100	0.8389797170280731
■	199	13	LSTM	512	128	0.05	100	0.838605991353371
■	320	13	LSTM	1024	256	0.05	100	0.8382258141295637
■	334	13	GRU	1024	512	None	100	0.838140482303067
■	279	13	GRU	512	256	0.05	100	0.8381249583390414
■	331	13	GRU	1024	256	0.05	None	0.8379533310023022
■	267	13	GRU	512	128	None	100	0.837781993880267

Version 2-0 top 25 hyperparametric configurations based on ORC. An interactive version of this chart is available on GitHub: https://sbhattacharyay.github.io/TILTomorrow/TILTomorrow_model_performance/v2-0/ORC_hiplot.html.

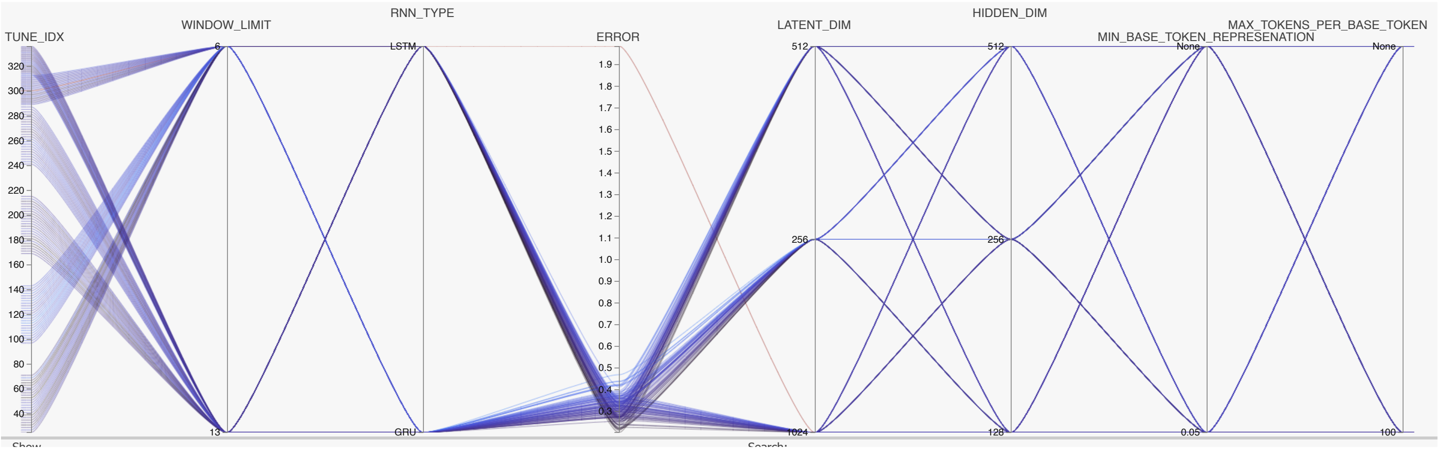

Version 2-0 HiPlots of macro-averaged calibration slope error. An interactive version of the HiPlot is available on GitHub: https://sbhattacharyay.github.io/TILTomorrow/TILTomorrow_model_performance/v2-0/thresh_calibration_hiplot.html.

TUNE_IDX	WINDOW_LIMIT	RNN_TYPE	LATENT_DIM	HIDDEN_DIM	MIN_BASE_TOKEN_REPRESENATION	MAX_TOKENS_PER_BASE_TOKEN	ERROR
53	6	LSTM	512	128	0.05	None	0.2027243284878944
65	6	LSTM	512	512	None	None	0.2128540506690903
39	6	LSTM	256	256	0.05	100	0.21734548483198873
181	13	LSTM	256	256	0.05	None	0.2190650489945436
63	6	LSTM	512	256	0.05	100	0.22024893048456684
177	13	LSTM	256	256	None	None	0.22203808385460866
31	6	LSTM	256	128	0.05	100	0.2264820278197573
320	13	LSTM	1024	256	0.05	100	0.22667236901330745
211	13	LSTM	512	512	None	100	0.23626360318497205
297	6	LSTM	1024	512	None	None	0.23730447320492137
175	13	LSTM	256	128	0.05	100	0.237418278366817
294	6	LSTM	1024	256	None	100	0.24011129666603126
67	6	LSTM	512	512	None	100	0.2434552211485323
35	6	LSTM	256	256	None	100	0.2499676164652886
191	13	LSTM	256	512	0.05	100	0.2516177026768438
267	13	GRU	512	128	None	100	0.2541600858933404
201	13	LSTM	512	256	None	None	0.2557812950950744
316	13	LSTM	1024	128	0.05	100	0.25905303624606385
183	13	LSTM	256	256	0.05	100	0.2599552967513904
33	6	LSTM	256	256	None	None	0.2641243560257474
57	6	LSTM	512	256	None	None	0.2657096651743324
291	6	LSTM	1024	128	0.05	None	0.2657932320234061
318	13	LSTM	1024	256	None	100	0.2683017451805711
207	13	LSTM	512	256	0.05	100	0.26929298783368893
71	6	LSTM	512	512	0.05	100	0.271057399569399

Version 2-0 top 25 hyperparametric configurations based on macro-averaged calibration slope error. An interactive version of this chart is available on GitHub: https://sbhattacharyay.github.io/TILTomorrow/TILTomorrow_model_performance/v2-0/thresh_calibration_hiplot.html.

Supplementary Methods S4. Calculation of Somers' D_{xy}.

Somers' D_{xy}, as proposed by Somers[R7] and Kim,[R8] is used as the primary metric for quantifying uncertainty in terms of explanation of the ordinal variation in next-day changes in TIL[(Basic)] from the variables in the CENTER-TBI dataset.

Carrying over the notation defined in Supplementary Methods S1, let us define $\epsilon_t^{(i)}$ as:

$$\epsilon_t^{(i)} = \sum_{l \in \{-1,0,1\}} l \cdot \pi_{l,t}^{(i)}$$

which corresponds to the expected direction of change in next-day TIL[(Basic)] from the last available score. At each of the days of performance assessment (i.e., $\forall t \in \{1,2,3,4,5,6,9,13\}$), the ϵ_t scores and the γ_t labels from across the assessment population are compiled into vectors:

$$\boldsymbol{\epsilon}_t = \left[\epsilon_t^{(1)}, \epsilon_t^{(2)}, \dots, \epsilon_t^{(N)}\right]^{\mathsf{T}}$$
$$\boldsymbol{\gamma}_t = \left[\gamma_t^{(1)}, \gamma_t^{(2)}, \dots, \gamma_t^{(N)}\right]^{\mathsf{T}}.$$

Somers' D_{xy} is defined by:

$$D_{xy,t} = \frac{\tau(\boldsymbol{\gamma}_t, \boldsymbol{\epsilon}_t)}{\tau(\boldsymbol{\gamma}_t, \boldsymbol{\gamma}_t)}$$

where τ is the Kendall's τ coefficient, defined for any two vectors $\mathbf{a}$ and $\mathbf{b}$:

$$\tau(\mathbf{b}, \mathbf{a}) = \frac{n_C(\mathbf{b}, \mathbf{a}) - n_D(\mathbf{b}, \mathbf{a})}{\binom{n}{2}}$$

where n is the length of $\mathbf{a}$ or $\mathbf{b}$, and $n_C(\mathbf{b}, \mathbf{a})$ is the number of concordant pairs between $\mathbf{a}$ and $\mathbf{b}$ and $n_D(\mathbf{b}, \mathbf{a})$ is the number of discordant pairs between $\mathbf{a}$ and $\mathbf{b}$.

Pairs between two vectors are concordant if both elements of the pair agree in rank. Between vectors $\boldsymbol{\gamma}_t$ and $\boldsymbol{\epsilon}_t$, a pair of patients $\{i,j\}$ is concordant if either $\epsilon_t^{(i)} > \epsilon_t^{(j)}$ and $\gamma_t^{(i)} > \gamma_t^{(j)}$ or $\epsilon_t^{(i)} < \epsilon_t^{(j)}$ and $\gamma_t^{(i)} < \gamma_t^{(j)}$. Between the vector $\boldsymbol{\gamma}_t$ and itself, a pair of patients $\{i,j\}$ is concordant if they have different endpoint classes. Pairs between two vectors are discordant if either element of the pair disagrees in rank. Between vectors $\boldsymbol{\gamma}_t$ and $\boldsymbol{\epsilon}_t$, a pair of patients $\{i,j\}$ is discordant if either $\epsilon_t^{(i)} > \epsilon_t^{(j)}$ and $\gamma_t^{(i)} < \gamma_t^{(j)}$ or $\epsilon_t^{(i)} < \epsilon_t^{(j)}$ and $\gamma_t^{(i)} > \gamma_t^{(j)}$. Between the vector $\boldsymbol{\gamma}_t$ and itself, there are no pairs that are discordant. Therefore, $\tau(\boldsymbol{\gamma}_t, \boldsymbol{\gamma}_t)$ is equivalent to the proportion of possible pairs of patients in the assessment population that have different endpoint classes at day t. This is considered a measure of the ordinal variation in the endpoint.[R4]

Let $n^{(\text{conc})}$ denote the number of concordant pairs between $\boldsymbol{\gamma}_t$ and $\boldsymbol{\epsilon}_t$, and let $n^{(\text{disc})}$ denote the number of discordant pairs between $\boldsymbol{\gamma}_t$ and $\boldsymbol{\epsilon}_t$. Let $n^{(\text{comp})}$ denote the number of pairs of patients within the assessment population with different endpoint classes (i.e., comparable pairs). The formula for Somers' D_{xy} can then be simplified to:

$$D_{xy,t} = \frac{n_C(\boldsymbol{\gamma}_t, \boldsymbol{\epsilon}_t) - n_D(\boldsymbol{\gamma}_t, \boldsymbol{\epsilon}_t)}{n_C(\boldsymbol{\gamma}_t, \boldsymbol{\gamma}_t)}$$
$$= \frac{n^{(\text{conc})} - n^{(\text{disc})}}{n^{(\text{comp})}}.$$

Somers' D_{xy} equals the ratio of the difference between the number of concordant pairs and number of discordant pairs to the total number of comparable pairs. Assuming there are no ties in $\epsilon_t^{(i)}$ between patients of different $\gamma_t^{(i)}$,

$$= \frac{n^{(\text{conc})} - \left(n^{(\text{comp})} - n^{(\text{conc})}\right)}{n^{(\text{comp})}}$$
$$= \frac{2n^{(\text{conc})} - n^{(\text{comp})}}{n^{(\text{comp})}}$$
$$= 2\frac{n^{(\text{conc})}}{n^{(\text{comp})}} - 1$$
$$= 2\left[\frac{n^{(\text{conc})}}{\sum_{\mathrm{l}=-1}^{0} \sum_{\mathrm{m}=\mathrm{l}+1}^{1} \left|\Pi_{\mathrm{l},t}\right| \left|\Pi_{\mathrm{m},t}\right|}\right] - 1$$
$$= 2\left[\frac{\sum_{\mathrm{l}'=-1}^{0} \sum_{\mathrm{m}'=\mathrm{l}'+1}^{1} \left|\Pi_{\mathrm{l}',t}\right| \left|\Pi_{\mathrm{m}',t}\right| \mathrm{c}_{\mathrm{l}'\mathrm{m}',t}}{\sum_{l=-1}^{0} \sum_{m=l+1}^{1} \left|\Pi_{l,t}\right| \left|\Pi_{m,t}\right|}\right] - 1$$

where $\Pi_{l,t} \subseteq \{1,2,\dots,N\}$ denotes the subset of indices of patients with $\gamma_t^{(i)} = l$ for each $l \in \{-1, 0, 1\}$ and $c_{l'm',t}$ denotes the pairwise c-index (i.e., area under the receiver operating characteristic curve [AUC]) between patients with $\gamma_t^{(i)} = l'$ and those with $\gamma_t^{(i)} = m'$. In other words, Somers' D_{xy} is equivalent to twice the prevalence-weighted average of pairwise c-indices minus one. Therefore, the feasible range of Somers' D_{xy} is 0 (or 0%) to 1 (or 100%). Somers' D_{xy} can also be interpreted as the **proportion of ordinal variation in the endpoint that can be explained by the variation in model output**.

Supplementary Methods S5. Explanation of model outputs with Shapley value estimations.

On an individual patient level, we estimated the contribution of specific variables towards trained model outputs with algorithmic approximations of Shapley values. Shapley values, developed originally for cooperative game theory,[R9] distribute a reward (or loss) amongst members of a team based on their positive or negative contributions. Now, suppose we represent a patient's feature values – in our case, tokens – as teammates, and we let the difference between a patient's model output and the average model output be the reward. Then, Shapley values can theoretically provide a window into how the model's output is affected by the values of specific features, regardless of the model's structure.

Shapley values

Suppose we have a trained, static version of a TILTomorrow model which only predicts next-day TIL$^{(\text{Basic})}$ on day one of ICU stay. Let M represent the total number of tokens stored in the embedding layer dictionary and let $\mathbf{x}^{(i)} \in \{0,1\}^M$ be a binary vector representing a patient's set of tokens for the first calendar day of ICU stay such that a 1 represents the existence of the corresponding dictionary token in the time window. The Shapley value of a token with index $j \in \{1,2,\ldots,M\}$ where $x_j^{(i)} = 1$ is defined as:

$$\phi_j^{(i)} = \sum_{S \subseteq \{1,2,\ldots,\mathrm{M}\} \setminus \{j\}} \frac{|S|!\,(M - |S| - 1)!}{M!} \left(v_{\mathbf{x}^{(i)}}(S \cup \{j\}) - v_{\mathbf{x}^{(i)}}(S) \right)$$

where S is a subset of tokens (i.e., coalition) for which the patient's true values are taken and $v_{\mathbf{x}^{(i)}}(S)$ is a function which calculates the marginal contribution of a coalition towards model output:

$$v_{\mathbf{x}^{(i)}}(S) = \int \ldots \int \hat{f}\left(\mathbf{x}^{(i)}\right) d\mathbb{T}_{\setminus S} - \mathbb{E}_{\mathbf{x}}\left[\hat{f}(\mathbf{x})\right]$$

where $\mathbb{T}_{\setminus S}$ is the token space excluding tokens in the coalition S, $\hat{f}$ is a function that returns the trained model output for a given token set $\mathbf{x}$, and $\mathbb{E}_{\mathbf{x}}\left[\hat{f}(\mathbf{x})\right]$ is the average model output. In other words, the Shapley value of a specific token equals its average marginal contribution across all possible coalitions. Coalitions are weighted by size to provide greater influence on a specific token's effect when it is closer to isolation (i.e., $|S| \to 0$) or the patient's true token set (i.e., $|S| \to M$). $v_{\mathbf{x}^{(i)}}(S)$ integrates out all the effects of tokens not in the given coalition and subtracts the average model output to return the marginal contribution of the coalition of variables towards model output. In this analysis, the chosen model output for Shapley value estimation is the expected next-day TIL$^{(\text{Basic})}$ score:

$$\omega_t^{(i)} = \sum_{k=0}^{4} k \cdot p_{k,t}^{(i)}$$

with notation defined in Supplementary Methods S1. Shapley values can be interpreted as **a token's contribution to the difference between an individual patient's model output and the population-average model output, given the patient's full set of tokens**.

However, Shapley values pose several practical challenges for our application. Direct Shapley value calculation is infeasible, as it would require iterating through up to 2^M (where $M \approx 30{,}000$) coalitions per patient. Moreover, in the sparse latent space of our embedding layer, integration over coalitions of tokens is not trivial. TILTomorrow is a dynamic modelling strategy, and Shapley values would have to be extended into the temporal dimension, further complicating the feasibility of their estimation.

KernelSHAP

The SHapley Additive exPlanations (SHAP) method, proposed by Lundberg *et al.*,[R10] has become a popular tool for estimating Shapley values with a linear model. Suppose we have a patient i with binary token vector $\mathbf{x}^{(i)}$. We are interested in understanding how the tokens contribute towards $\omega^{(i)}$, and we designate $\hat{f}: \{0,1\}^{M \times 1} \to \mathbb{R}_{[0,4]}$ as the trained model function:

$$\hat{f}\left(\mathbf{x}^{(i)}\right) = \omega^{(i)}.$$

For this specific case, SHAP intends to learn a linear explanation function $g^{(i)}$ which maps a binary coalition vector $\boldsymbol{\zeta}^{(i)} \in \{0,1\}^{M \times 1}$ – which specifies the elements of $\mathbf{x}^{(i)}$ that are maintained in the coalition – to a value that approximates $\hat{f}\left(\mathbf{x}^{(i)}\right)$ when $\boldsymbol{\zeta}^{(i)} \approx 1$. Then, $g^{(i)}$ can be represented as:

$$g^{(i)}\left(\boldsymbol{\zeta}^{(i)}\right) = \phi_0^{(i)} + \sum_{j=1}^{M} \phi_j^{(i)} \zeta_j^{(i)},$$

i.e., the sum of Shapley values $\phi_j^{(i)}, \forall j \in \{1,2,\dots,M\}$.

Lundberg *et al*. proposed the KernelSHAP algorithm which estimates the Shapley values by sampling coalition vector data and fitting a weighted linear regression model.[R10] First, we need to define a mapping function $h_{\mathbf{x}^{(i)}}(\boldsymbol{\zeta}^{(i)})$ which transforms the coalition assignments from $\boldsymbol{\zeta}^{(i)}$ to the space of $\mathbf{x}^{(i)}$. For our application, this is quite simple, since $\mathbf{x}^{(i)}$ is itself a binary vector:

$$h_{\mathbf{x}^{(i)}}(\boldsymbol{\zeta}^{(i)}) = \mathbf{x}^{(i)} \odot \boldsymbol{\zeta}^{(i)} + (1 - \boldsymbol{\zeta}^{(i)}) \odot \mathbf{b}$$

where $\mathbf{b} \in \{0,1\}^{M\times 1}$ is a baseline vector which replaces each out-of-coalition value in $\mathbf{x}^{(i)}$ with a value from elsewhere. In this work, we used replacement with the mode of that index across the training set. Then, the algorithm samples Z different combinations of $\boldsymbol{\zeta}^{(i)}$ (i.e., coalitions) and calculates $\hat{f}\left(h_{\mathbf{x}^{(i)}}(\boldsymbol{\zeta}^{(i)})\right)$ for each one. In our applications, we constrained coalition sampling so that: (1) only indices corresponding to a token represented in $\mathbf{x}^{(i)}$ could be perturbed, i.e., only sampling from $\left\{j \in \{1,2,\dots,M\}: x_j^{(i)} = 1\right\}$, and (2) sampling would exhaust coalitions of large and small sizes first before working towards middle-size coalitions until Z samples were obtained. This is motivated by the Shapley value equation, which weighs coalitions of small and large sizes more heavily. After all coalitions were sampled and combined into set $\mathcal{Z} = \left\{\boldsymbol{\zeta}_1^{(i)}, \boldsymbol{\zeta}_2^{(i)}, \dots, \boldsymbol{\zeta}_Z^{(i)}\right\}$, Shapley values were estimated by optimising the following loss function:

$$\begin{aligned} \ell^{(i)}(\hat{f}, g^{(i)}, \pi_{\mathbf{x}^{(i)}}) &= \sum_{\boldsymbol{\zeta}_j^{(i)} \in \mathcal{Z}} \left[\hat{f}\left(h_{\mathbf{x}^{(i)}}(\boldsymbol{\zeta}^{(i)})\right) - g^{(i)}(\boldsymbol{\zeta}_j^{(i)})\right]^2 \pi_{\mathbf{x}^{(i)}}(\boldsymbol{\zeta}_j^{(i)}) \\ &= \sum_{\boldsymbol{\zeta}_j^{(i)} \in \mathcal{Z}} \left[\hat{f}\left(h_{\mathbf{x}^{(i)}}(\boldsymbol{\zeta}^{(i)})\right) - \phi^{(i)\top}\boldsymbol{\zeta}_j^{(i)}\right]^2 \pi_{\mathbf{x}^{(i)}}(\boldsymbol{\zeta}_j^{(i)}) \end{aligned}$$

where $\pi_{\mathbf{x}^{(i)}}$ is the kernel set to achieve similar weighting as the Shapley equation:

$$\pi_{\mathbf{x}^{(i)}}(\boldsymbol{\zeta}_j^{(i)}) = \frac{(M-1)}{\binom{M}{|\boldsymbol{\zeta}_j^{(i)}|}\left|\boldsymbol{\zeta}_j^{(i)}\right|\left(M - \left|\boldsymbol{\zeta}_j^{(i)}\right|\right)}.$$

TimeSHAP and ΔTimeSHAP

TimeSHAP is a temporal extension of the KernelSHAP algorithm proposed by Bento *et al.*[R11] for efficient and multi-level model output explanation. Like several other temporal extensions of KernelSHAP, TimeSHAP estimates the contribution of tokens and time windows before a certain model output. However, TimeSHAP also groups combinations of tokens and time windows in meaningful ways to enhance the feasibility and focus of KernelSHAP. This starts with a temporal coalition pruning algorithm.

Temporal coalition pruning:

TimeSHAP starts by finding a point back in time before which tokens have a negligible effect on the current model output. Let the binary matrix $\mathbf{X}^{(i)} \in \{0,1\}^{M\times\mathcal{T}^{(i)}}$ be the tokenised representation of a patient's ICU record, where each row represents a token in the training set dictionary and each column represents a calendar day in the patient's ICU stay. Suppose we are interested in explaining the output of a trained dynamic model $(\hat{f})$ at the last time window, $\mathcal{T}^{(i)}$. The temporal coalition pruning algorithm first groups all the tokens at $\mathcal{T}^{(i)}\left(\mathbf{X}_{:,\mathcal{T}^{(i)}}^{(i)}\right)$ as one "feature" and groups all the tokens from time $\{1,2,\dots,\mathcal{T}^{(i)} - 1\}\left(\mathbf{X}_{:,1:\mathcal{T}^{(i)}-1}^{(i)}\right)$ as another feature, and runs KernelSHAP on just these two features ($2^2 = 4$ total coalitions). Then, the algorithm pushes back one step in time, groups tokens from $\{\mathcal{T}^{(i)} - 1, \mathcal{T}^{(i)}\}$ and $\{1,2,\dots,\mathcal{T}^{(i)} - 2\}$ into two separate features $\left(\mathbf{X}_{:,\mathcal{T}^{(i)}-1:\mathcal{T}^{(i)}}^{(i)} \text{ and } \mathbf{X}_{:,1:\mathcal{T}^{(i)}-2}^{(i)}\right)$, and runs KernelSHAP again. This process is iteratively repeated, pushing back one step at a time, until the estimated Shapley value corresponding to the block of earlier time windows falls below a certain tolerance criterion, $\eta \in \mathbb{R}_{>0}$. Let $\mathcal{T}^{(i)} - l$ represent the time window threshold at which this happens. Then, tokens of at time windows $\{1,2,\dots,\mathcal{T}^{(i)} - l\}$ are pruned together as one feature, thereby reducing the number of possible coalitions in future KernelSHAP runs. Our selected criterion value was $\eta = 0.025$ based on the recommendations of the original TimeSHAP report.[R11]

Token- and time-level explanations:

Once the pruned time windows $\{1,2,\dots,\mathcal{T}^{(i)} - l\}$ are lumped into a single feature, TimeSHAP then groups each of the tokens across the remaining time windows (i.e., the recent past) as features. In other words, each row of $\mathbf{X}_{:,\mathcal{T}^{(i)}-l+1:\mathcal{T}^{(i)}}^{(i)}$ is grouped

as a feature, and these M features (along with the pruned time windows as a single feature) are fed into KernelSHAP to estimate the token-level Shapley values. Thereafter, each of the l remaining time windows – i.e., each column of $\mathbf{X}^{(i)}_{:,\mathcal{T}^{(i)}-l+1:\mathcal{T}^{(i)}}$ – is grouped as a feature, and KernelSHAP is used to estimate the time-level Shapley values for each of them. TimeSHAP also permits estimation of cell-level Shapley values (i.e., at specific combinations of tokens and time windows), but we did not calculate these values for our analyses.

ΔTimeSHAP:
The ordinal endpoint of our dynamic model is itself a dynamic variable. Therefore, we were interested in using TimeSHAP to uncover features associated with changes in next-day $\text{TIL}^{(\text{Basic})}$.

Let $t^* \in \{1,2,\dots,\mathcal{T}^{(i)}\}$ denote a day at which the next-day $\text{TIL}^{(\text{Basic})}$ score is different from the last available $\text{TIL}^{(\text{Basic})}$ score. With the TimeSHAP algorithm, we estimated Shapley values (ϕ) for each token $j \in \{1,2,\dots,M\}$ in two days directly preceding a day-to-day change in $\text{TIL}^{(\text{Basic})}$ (i.e., $\{t^*, t^*-1\}$) to calculate:

$$\Delta\phi^{(i)}_{j,t^*} = \phi^{(i)}_{j,t^*} - \phi^{(i)}_{j,t^*-1},$$

which we refer to as the token's ΔTimeSHAP value. If a token did not exist in the window of either of the two days, then its ϕ value for that day was zero. Assuming the population-average model output $\left(\mathbb{E}_{\mathbf{X}}\left[\widehat{f_\omega}(\mathbf{X})\right]\right)$ does not change substantially between the two days, ΔTimeSHAP values can be interpreted as **a token's contribution to the difference in an individual patient's model output over the two days directly preceding the change in $\text{TIL}^{(\text{Basic})}$, given the patient's full set of tokens**. If a variable had a positive (or negative) ΔTimeSHAP value, it was associated with an increased likelihood of escalation (or de-escalation) in next-day treatment intensity. Moreover, since the calculation of ΔTimeSHAP values required two days of information before the change in $\text{TIL}^{(\text{Basic})}$, we only calculated the variable contributions to day-to-day changes in $\text{TIL}^{(\text{Basic})}$ that occurred after day two of ICU stay.

SUPPLEMENTARY REFERENCES

R1. Guo C, Pleiss G, Sun Y, Weinberger KQ. On Calibration of Modern Neural Networks. In: Proceedings of the 34th International Conference on Machine Learning. PMLR, 2017: 1321–30.

R2. Tsamardinos I, Greasidou E, Borboudakis G. Bootstrapping the out-of-sample predictions for efficient and accurate cross-validation. *Mach Learn* 2018; **107**: 1895–922.

R3. Mnich K, Kitlas Golińska A, Polewko-Klim A, Rudnicki WR. Bootstrap Bias Corrected Cross Validation Applied to Super Learning. In: Krzhizhanovskaya VV, Závodszky G, Lees MH, *et al.*, eds. Computational Science – ICCS 2020. Cham: Springer International Publishing, 2020: 550–63.

R4. Van Calster B, Van Belle V, Vergouwe Y, Steyerberg EW. Discrimination ability of prediction models for ordinal outcomes: Relationships between existing measures and a new measure. *Biom J* 2012; **54**: 674–85.

R5. Van Calster B, Nieboer D, Vergouwe Y, De Cock B, Pencina MJ, Steyerberg EW. A calibration hierarchy for risk models was defined: from utopia to empirical data. *J Clin Epidemiol* 2016; **74**: 167–76.

R6. Bhattacharyay S, Caruso PF, Åkerlund C, *et al.* Mining the contribution of intensive care clinical course to outcome after traumatic brain injury. *Npj Digit Med* 2023; **6**: 1–11.

R7. Somers RH. A New Asymmetric Measure of Association for Ordinal Variables. *Am Sociol Rev* 1962; **27**: 799–811.

R8. Kim J-O. Predictive Measures of Ordinal Association. *Am J Sociol* 1971; **76**: 891–907.

R9. Shapley LS. A Value for n-Person Games. In: Contributions to the Theory of Games II. Princeton University Press, 1953: 307–18.

R10. Lundberg SM, Lee S-I. A Unified Approach to Interpreting Model Predictions. In: Guyon I, Luxburg UV, Bengio S, *et al.*, eds. Advances in Neural Information Processing Systems 30. NIPS, 2017: 4765–74.

R11. Bento J, Saleiro P, Cruz AF, Figueiredo MAT, Bizarro P. TimeSHAP: Explaining Recurrent Models through Sequence Perturbations. In: Proceedings of the 27th ACM SIGKDD Conference on Knowledge Discovery & Data Mining. New York, NY, USA: Association for Computing Machinery, 2021: 2565–73.
